# Supplementary material for: Chemical interplay and complementary adaptative strategies toggle bacterial antagonism and co-existence
Source: Cell Rep. 2021 Jul 27;36(4):109449. doi: 10.1016/j.celrep.2021.109449 (PMC8333196; doi:10.1016/j.celrep.2021.109449)
Supplement: Document S1. Figures S1–S9 and Tables S3, S4, S6, and S7 [file mmc1.pdf]

Cell Reports, Volume *36*

Supplemental information

Chemical interplay and complementary adaptative strategies toggle bacterial antagonism and co-existence

Carlos Molina-Santiago, David Vela-Corcía, Daniel Petras, Luis Díaz-Martínez, Alicia Isabel Pérez-Lorente, Sara Sopeña-Torres, John Pearson, Andrés Mauricio Caraballo-Rodríguez, Pieter C. Dorrestein, Antonio de Vicente, and Diego Romero

Supplementary data

Supplementary Figures

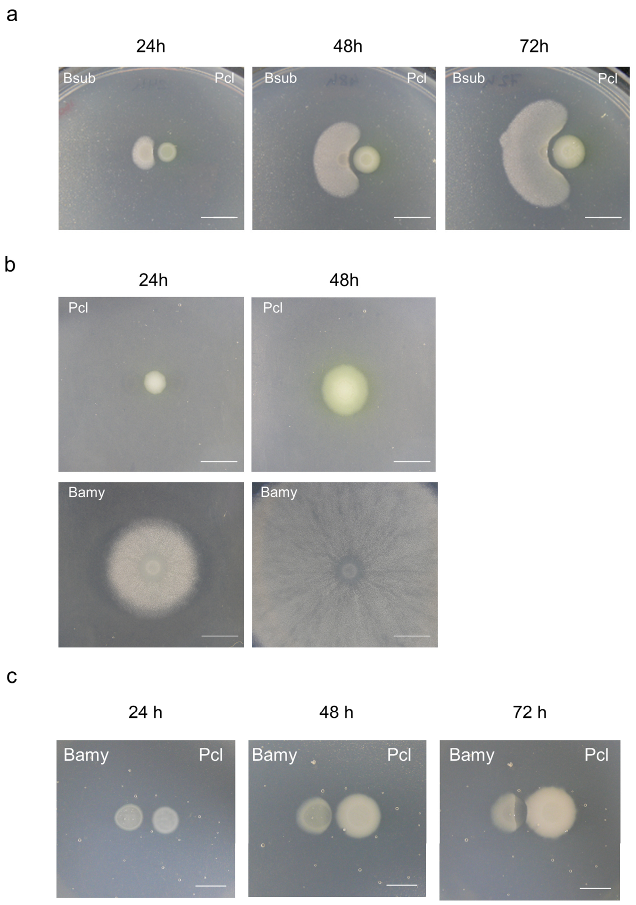

Figure S1. Pcl inhibits *B. subtilis* 3610 on King's B medium and Bamy on M9+glycerol. Related to Figure 1. a) Pairwise interaction time-lapse between *B. subtilis* 3610 (left) and Pcl (right) in King's B medium at 24, 48, and 72 h. Scale = 10 mm. b) Single colony growth of Pcl (top) and Bamy (bottom) on King's B medium at 24 and 48 h. c) Pairwise interaction time-lapse between Bamy (left) and Pcl (right) in M9 medium supplemented with glycerol as sole carbon source at 24, 48, and 72 h. Scale = 10 mm.

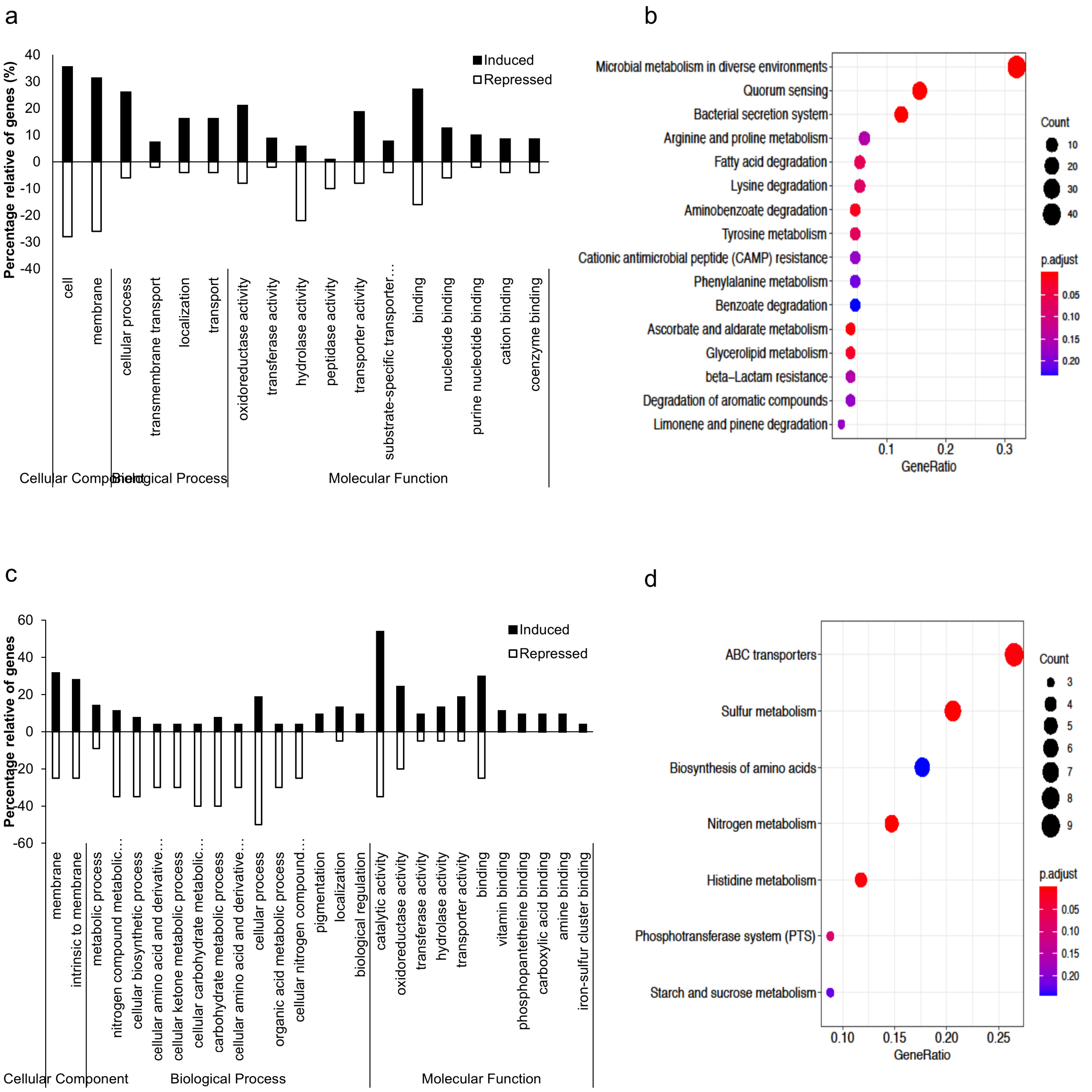

Figure S2. Transcriptomic changes during the interaction of Pcl with Bamy at 24 h. Related to Figure 2. a) GO terms of differentially expressed genes in Pcl. Black bars indicate induced GO terms and empty bars indicate repressed GO terms. b) KEGG pathways differentially expressed during the interaction in Pcl compared with control samples. c) KEGG pathways differentially expressed during the interaction in Bamy compared with control samples. d) GO terms of differentially expressed genes in Bamy. Black bars indicate induced GO terms and empty bars indicate repressed GO terms.

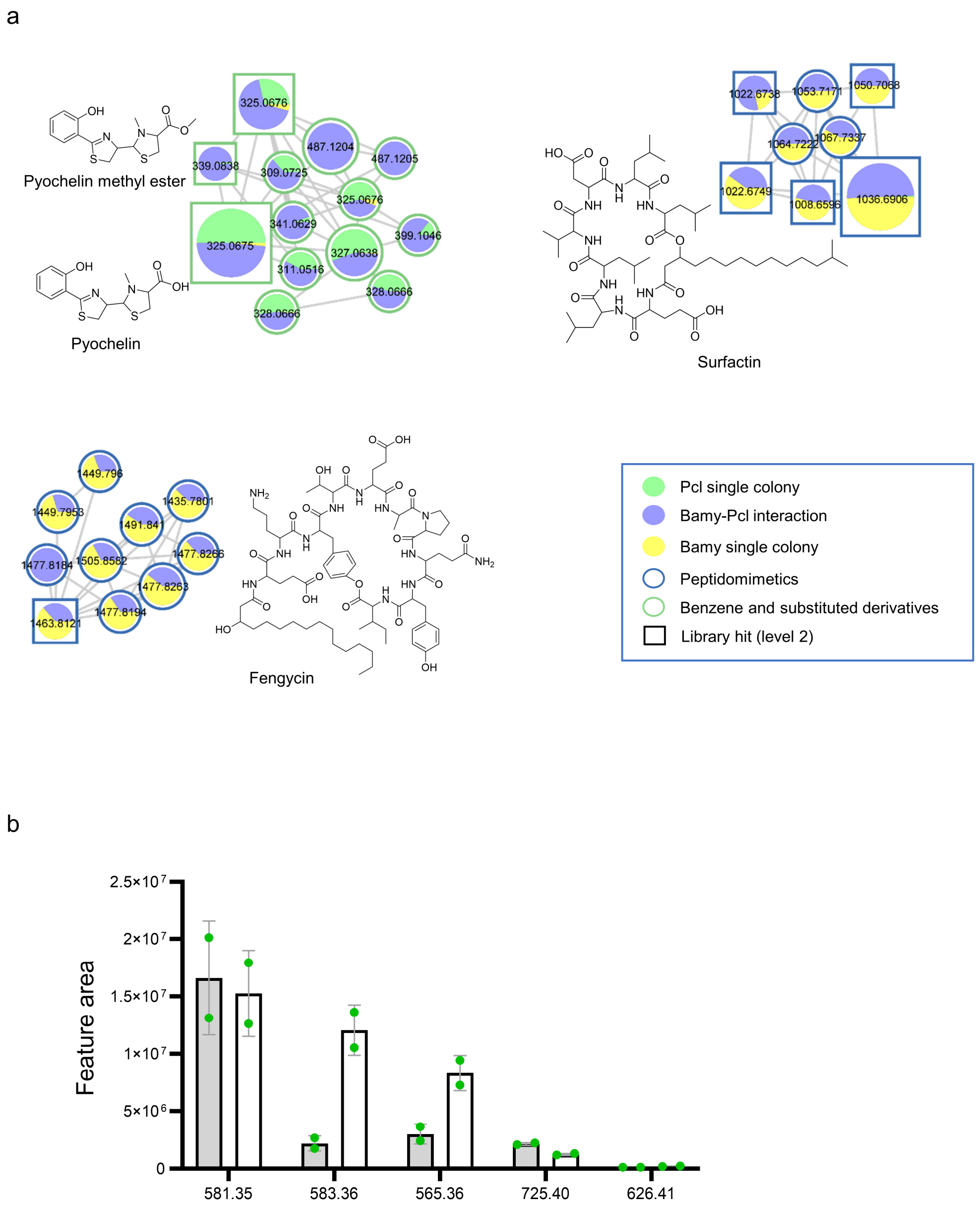

Figure S3. a) Molecular families of pyochelin, surfactin and fengycin detected from Bamy and Pcl growing alone and in interaction. Related to Figure 2. Results shown were obtained by mass spectrometry using LC-MS/MS and feature based molecular networking. Each metabolite is represented by a circle and they are connected according to the mass fragmentation patterns. The chemical structures of the annotated features are based on spectral matches in GNPS libraries representative of specific molecular families. Border

colors indicate ClassyFire classification. The sizes of the compounds are directly related to their abundance in the metabolome. Squares indicate a library hit level 2 through GNPS, and circles indicate unknown compounds based on GNPS searches. b) Bar plots show the quantification of the relative abundance of the selected molecules in Bamy growing alone and in interaction with Pcl. Grey bars represent the feature area of metabolites in Bamy single colonies, while empty bars represent the feature area of metabolites produced by Bamy during the interaction with Pcl. Statistical analysis done using t-test. Error bars indicate SD, *n* = 2.

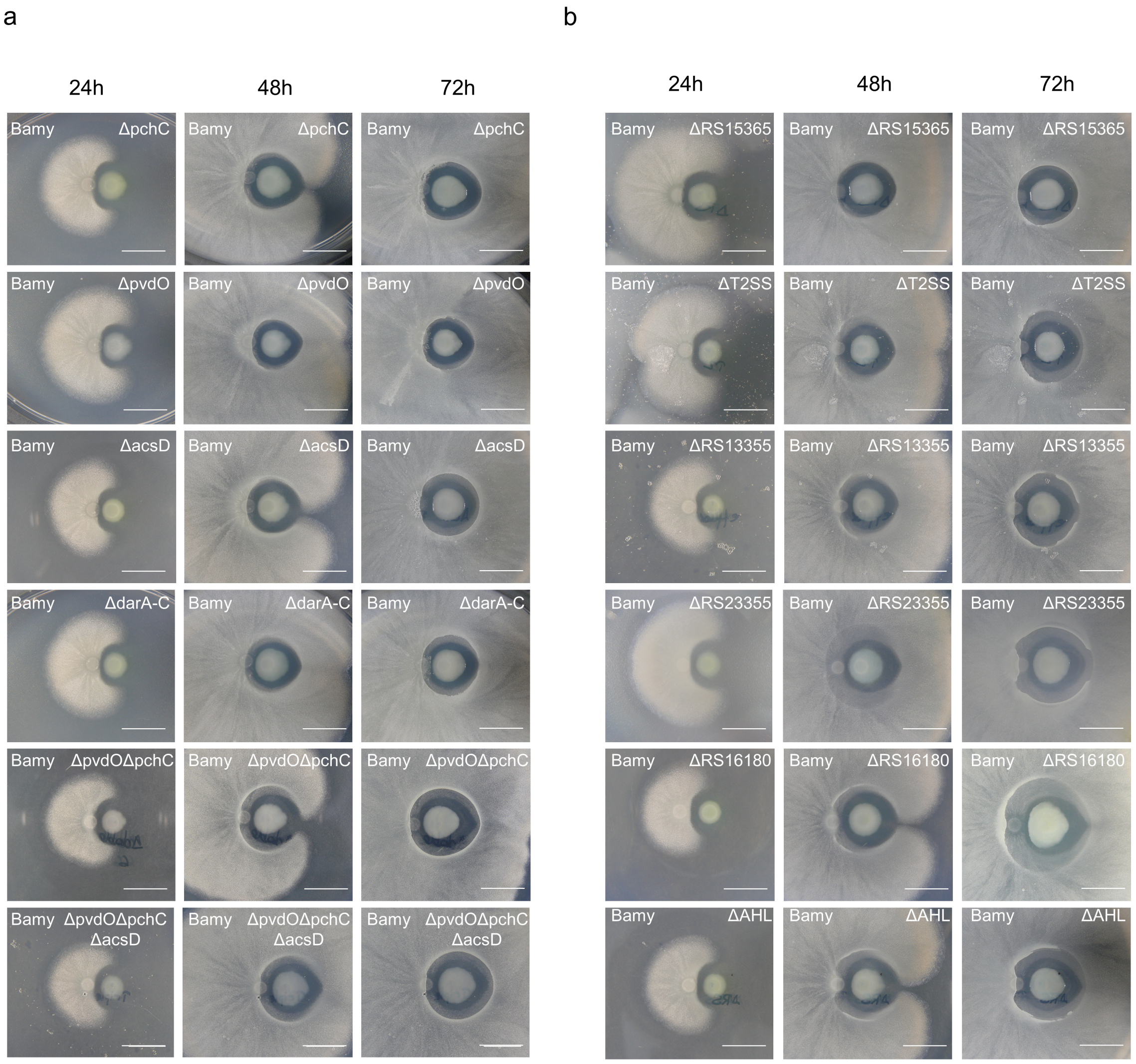

Figure S4. a) Time-course pairwise interactions between Bamy and Pcl mutants involving secondary metabolites on King's B medium at 24, 48, and 72 h. Related to Figure 2. Scale = 10 mm. ΔpvdO = pyoverdine mutant; ΔpchC = pyochelin mutant; ΔacsD = achromobactin mutant; ΔdarA-C = HPR mutant; ΔpvdOΔpchC = double mutant in pyoverdine and pyochelin; ΔpvdOΔpchCΔacsD = triple mutant in pyoverdine, pyochelin, and achromobactin. b) Time-course pairwise interactions between Bamy and Pcl mutants involving secondary metabolites, T2SS, and efflux pumps on King's B medium at 24, 48, and 72 h. Scale = 10 mm. ΔAHL = acyl-homoserine lactone mutant; ΔT2SS = type II secretion system mutant.

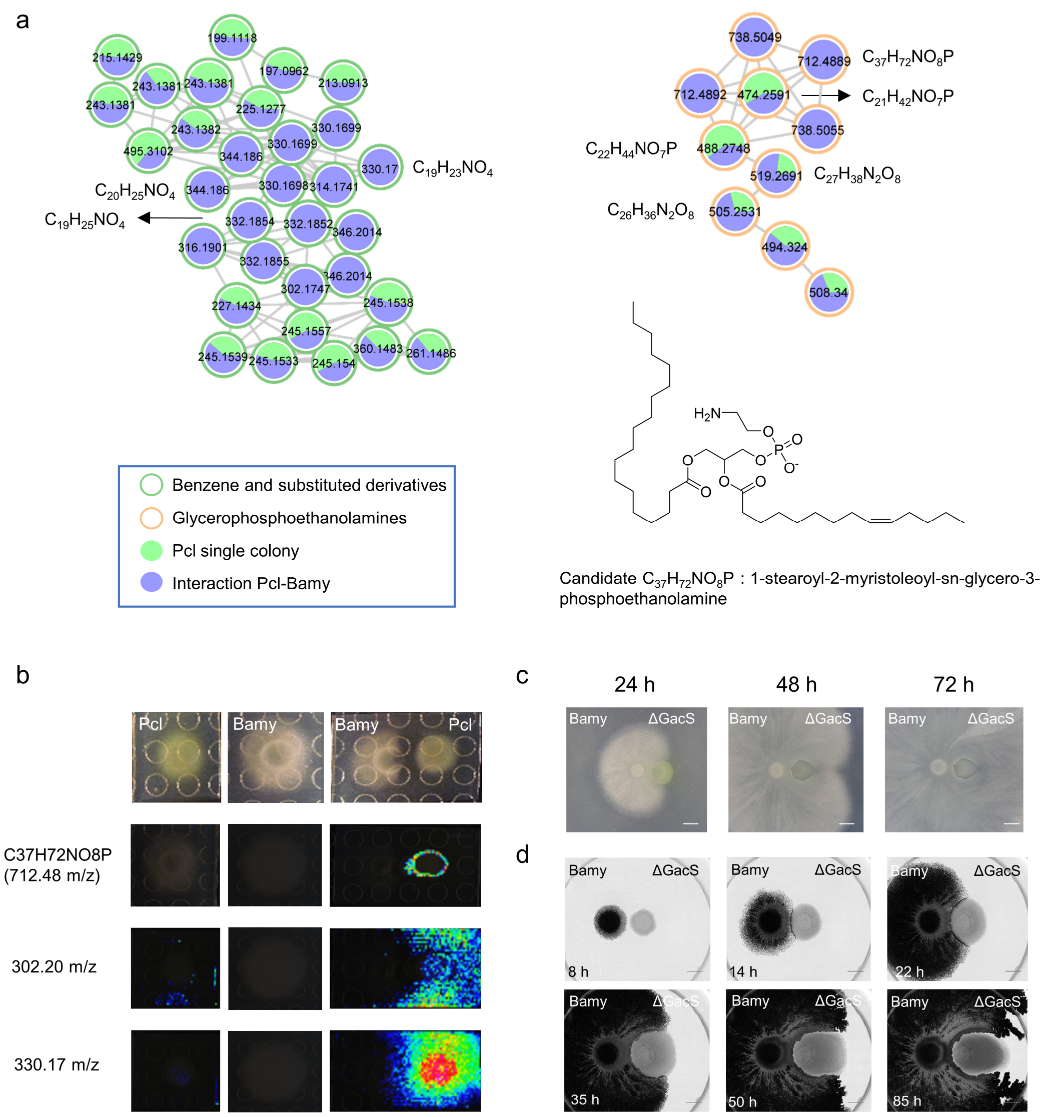

Figure S5. Candidate Pcl metabolites responsible for Bamy inhibition. Related to Figure 2. a) Molecular families of Pcl secondary metabolites mostly detected during the interaction with Bamy. Chemical structure of annotated features based on SIRIUS analyses, as representative of these molecular families. Border color indicates ClassyFire classification. The sizes of the compounds are directly related to their abundance in the metabolome. Squares indicate a library hit level 2 in GNPS, while circles indicate unknown compounds based on GNPS. b) MALDI-TOF-MSI heatmaps showing the spatial distribution of

representative metabolites indicated in panel a with m/z 712.48, 302.20 and 330.17. c) Pairwise interaction time lapse between Bamy (left) and *ΔGacS* (right) on King's B media at 24, 48, and 72 h. Scale = 5 mm. d) Time-lapse microscopy of the pairwise interaction between Bamy (left) and *ΔGacS* (right) during 85 h. Scale= 2 mm.

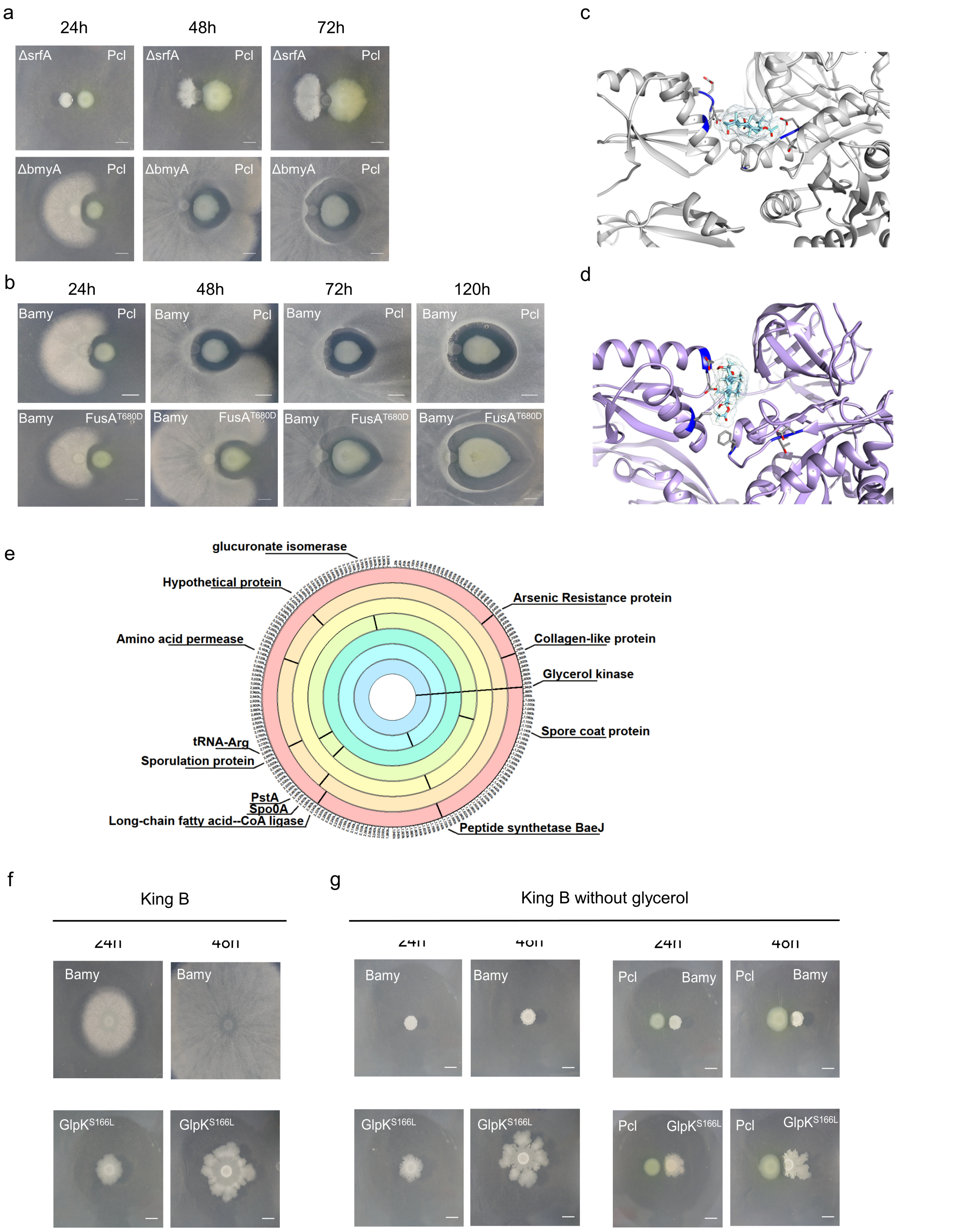

Figure S6. Related to Figures 3 and 4. a) Pairwise interactions between Pcl and Bamy mutants related to the secondary metabolites surfactin (*ΔsrfA*) (top) and bacillomycin (*ΔbmyA*) (bottom). Scale = 5 mm. b) Pairwise interactions between Bamy and Pcl WT (top)

or FusA^{T680D} (bottom) on King's B medium at 24, 48, 72 and 120 h. Scale = 5 mm. c and d) Molecular docking between fusidic acid and (c) FusA or (d) FusA^{T680D} showing differences in the binding pocket. e) Circular representation of the point mutations found in the genomes of Bamy clones with *glpK* mutations. Each ring represents one sequenced Bamy clone. f) and g) Growth differences in Bamy strains on King's B medium with and without glycerol. f) Single Bamy and GlpK^{S166L} colonies growing on King's B medium at 24 and 48 h. g) Single Bamy and GlpK^{S166L} colonies and their interactions with Pcl growing on King's B medium without glycerol at 24 and 48 h.

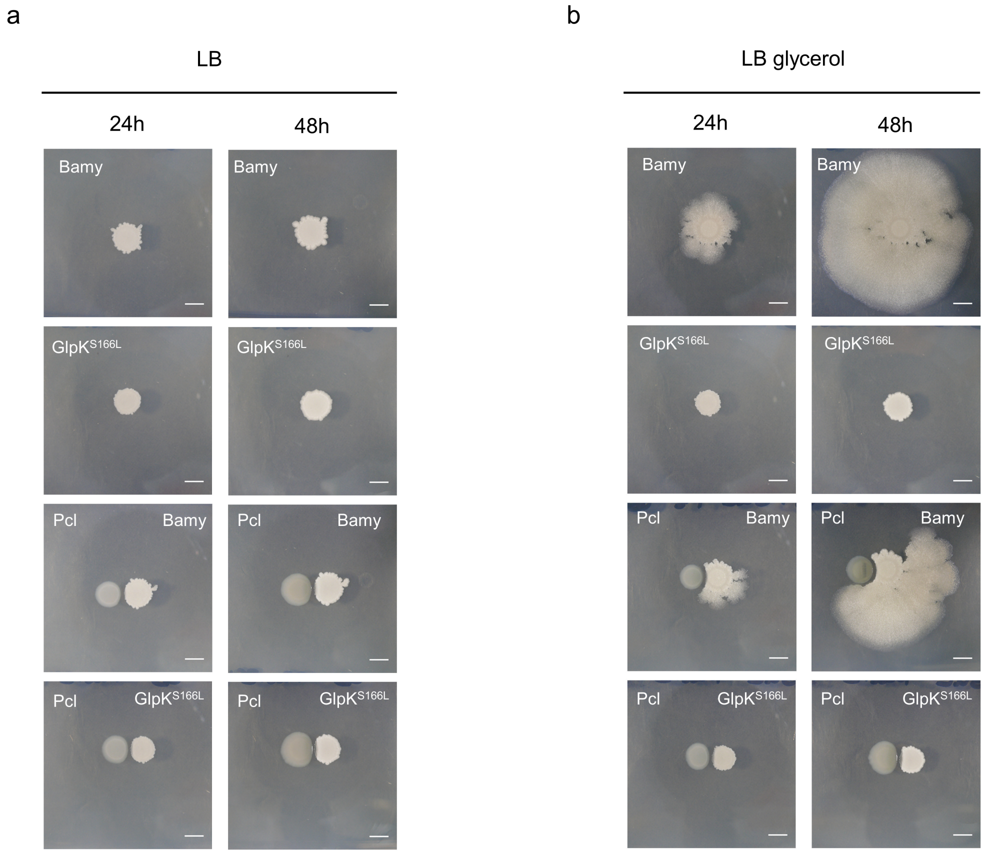

Figure S7. Growth differences in Bamy strains on LB medium with and without glycerol. Related to Figure 5. a) Single Bamy and GlpK^{S166L} colonies and their interactions with Pcl growing on LB B medium at 24 and 48 h. b) Single Bamy and GlpK^{S166L} colonies and their interactions with Pcl growing on LB medium supplemented with glycerol at 24 and 48 h. Scale = 5 mm.

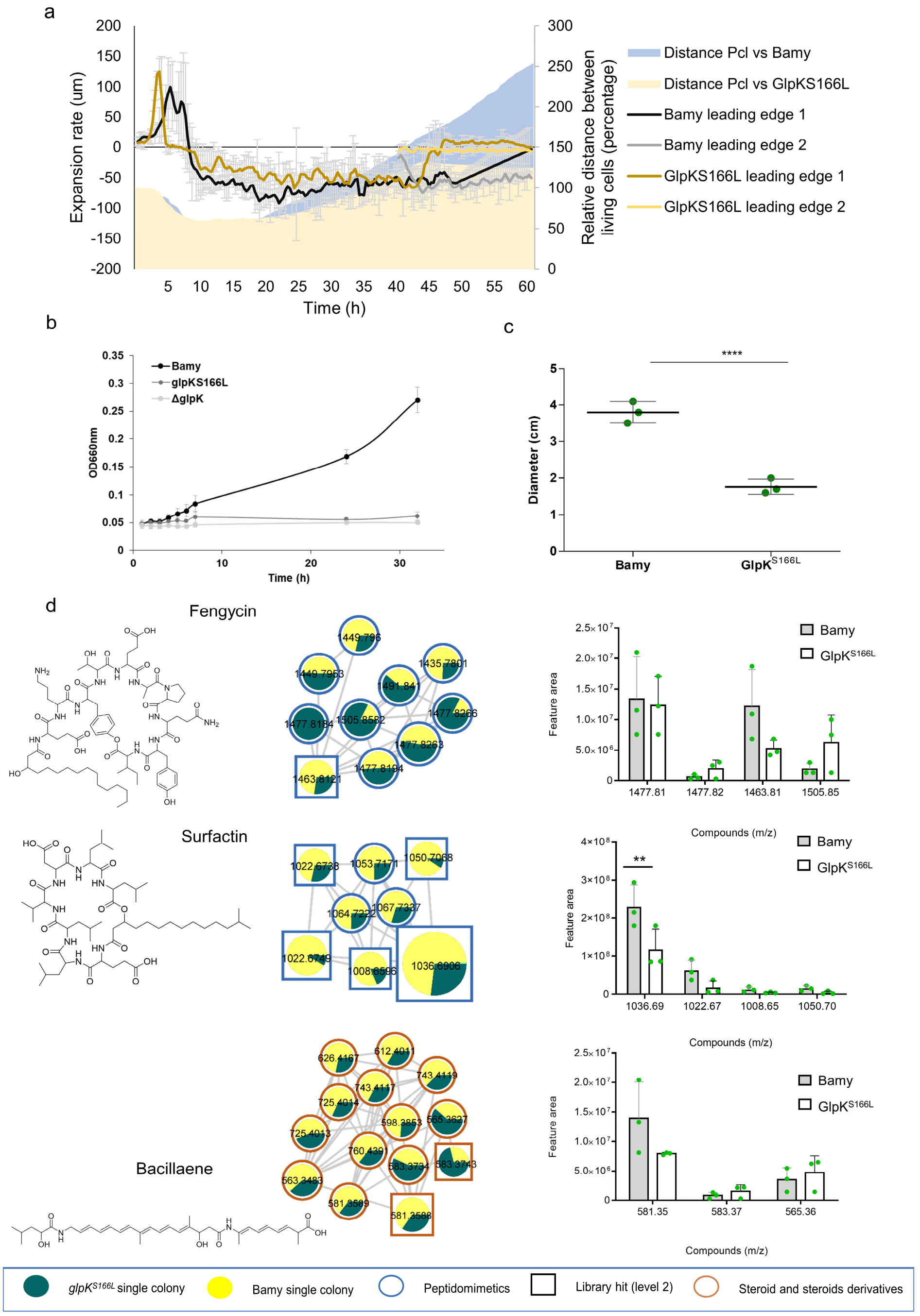

Figure S8. Phenotypic changes in a GlpK^{S166L} mutant. Related to Figures 5 and 6. a) Expansion rates and distance of the GlpK^{S166L} and Bamy leading edges during the interaction with Pcl. Brown and yellow lines represent GlpK^{S166L} leading edges 1 and 2. Black

and grey lines represent Bamy leading edges 1 and 2. Blue area represents the distance between the Bamy and Pcl populations during the entire interaction while the light orange area represents the distance between the GlpK^{S166L} and Pcl populations. Error bars represent SD. b) Growth curves of Bamy (black dots), GlpK^{S166L} (dark grey dots) and ΔglpK (grey dots) in M9 supplemented with 5 mM glycerol. c) Swarming motility reduction in a GlpK^{S166L} mutant. The plot represents the diameter of the colony. Statistical analysis done using t-test. *****P*-value < 0.0001. Error bars represent SD, *n* = 3. d) Metabolomic changes found in GlpK^{S166L} in comparison with Bamy. Molecular families of the secondary metabolites fengycin, surfactin, and bacillaene. The chemical structures of annotated features are based on spectral matches to GNPS libraries as representative of these molecular families. Border color indicates ClassyFire classification. Yellow color represents relative abundance in Bamy, while dark green color represents relative abundance in GlpK^{S166L}. Bar plots show the quantification of the relative abundance of the selected molecules in the interactions in Bamy and GlpK^{S166L} strains. Grey bars represent Bamy feature area of metabolites, while empty bars represent GlpK^{S166L} feature area of metabolites. Statistical analysis done using t-test. ***P*-value < 0.01. Error bars represent SD, *n* = 23.

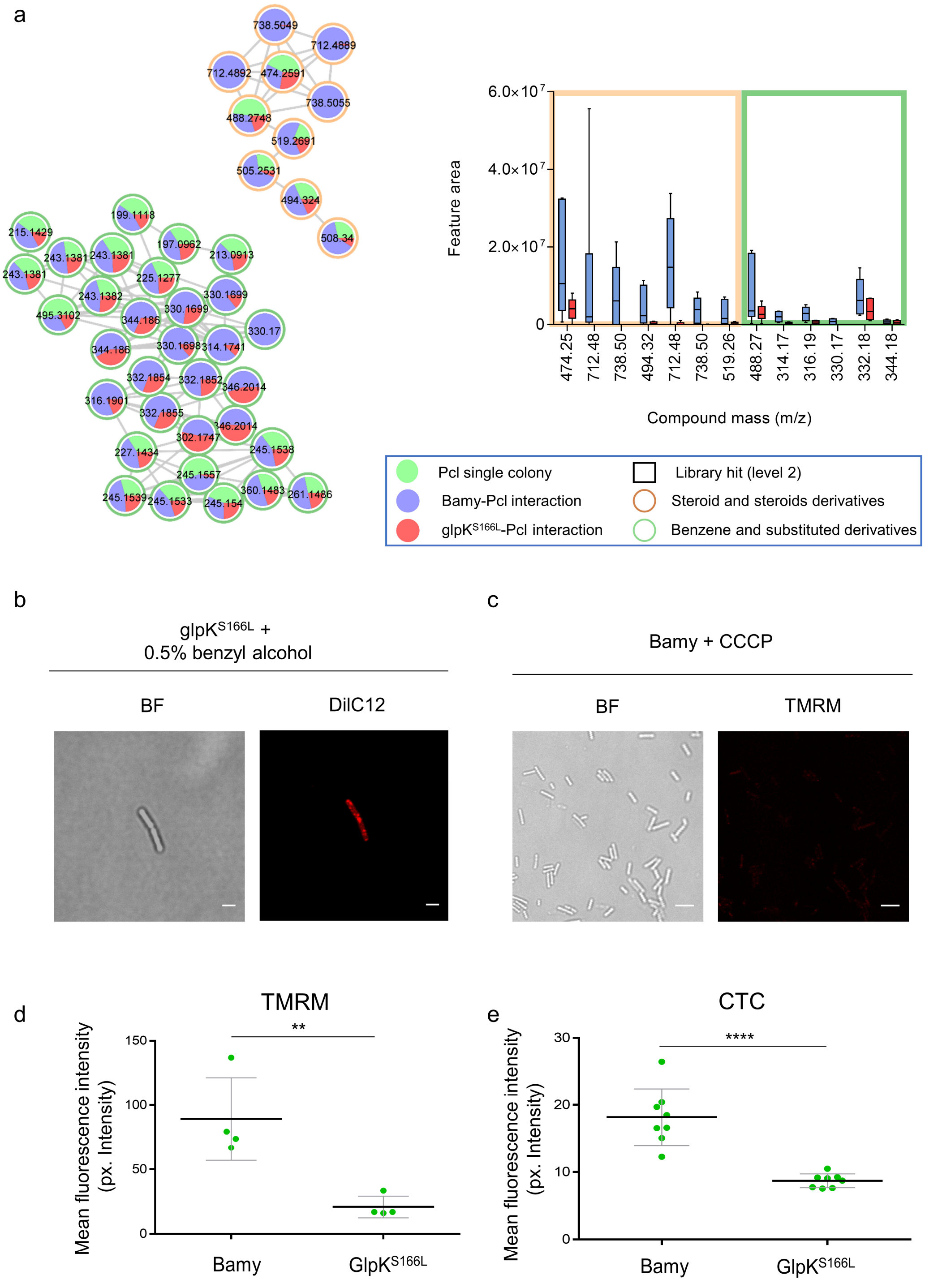

Figure S9. Metabolomic changes found in GlpK^{S166L} during the interaction with Pcl in comparison with the interaction of Bamy with Pcl. Related to Figure 6. a) Changes in the abundance of candidate molecular families of secondary metabolites produced by Pcl during

the interaction with Bamy and GlpK^{S166L}. The chemical structures of annotated features are based on spectral matches to GNPS libraries, as representative of these molecular families. Border color indicates ClassyFire classification. Right panel represents the quantification of the relative abundance of the selected molecules during Pcl-Bamy and Pcl-GlpK^{S166L} interactions. n = 6. Statistical test 2-way ANOVA. b) Membrane staining of GlpK^{S166L} with (left panel) DiIC12 dye using 0.5% benzyl alcohol as positive control (scale = 2 μm). c) TMRM positive control experiment of Bamy supplemented with 20 μM CCCP (scale 5 μm). d) Quantification of the TMRM signal in Bamy and GlpK^{S166L}. n = 4. e) Quantification of the CTC signal in Bamy and GlpK^{S166L} cells. Error bars represent the SEM, n = 8. Statistical significance in the TMRM and CTC experiments was assessed via two-tailed independent t-tests at each time-point (***P-value* < 0.01).

Supplementary Tables

Table S3. Metabolites purified from a Pcl culture with MIC activity against Bamy. Related to Figure 2.

**Mass (m/z)**	**Compound**	**MIC Bamy (µg/ml)**
237.18	2-Hexyl-5-propyl-rescorcinol (HPR)	40
239.2	unknown	62.5
339.08	Pyochelin methyl ester	250
444.22	unknown	250

Table S4. MIC of PcI and *fusA* mutants to aminoglycoside antibiotics kanamycin (Km) and gentamicin (Gm), and to fusidic acid. Related to Figure 4.

	**MIC (µg/ml)**		
	**Km**	**Gm**	**Fusidic acid**
PcI	4.5	2	3000
fusA^{T680D}	9	20	12000
fusA^{K366N}	9	20	3000

Table S6. Strains used in this study. Related to STAR Methods.

Strain	***Genotype***	***Reference***
***B. amyloliquefaciens* FZB42**	Wild type	*Bacillus* Genetic Stock Center (BGSC)
***B. amyloliquefaciens* FZB42**	glpK::km	This study
***B. amyloliquefaciens* FZB42**	Spontaneous mutant. *glpK*$^{F38S,\ W52stop,\ G254A}$	This study
***B. amyloliquefaciens* FZB42**	*Spontaneous mutant. glpK*$^{G147R,\ Q421R}$	This study
***B. amyloliquefaciens* FZB42**	*Spontaneous mutant. glpK*^{W355R}	This study
***B. amyloliquefaciens* FZB42**	*Spontaneous mutant. glpK*^{S166L}	This study
***B. amyloliquefaciens* FZB42**	*Spontaneous mutant. glpK*$^{+}$	This study
***B. amyloliquefaciens* FZB42**	*Spontaneous mutant. glpK*$^{G162R,\ W485stop}$	This study
***B. amyloliquefaciens* FZB42**	*Spontaneous mutant. glpK*$^{Q246-,\ G265S}$	This study
***B. amyloliquefaciens* FZB42**	Wild type, amyE:: Pveg-yfp	This study
***B. amyloliquefaciens* FZB42**	ΔfenA	*Bacillus* Genetic Stock Center (BGSC)
***B. amyloliquefaciens* FZB42**	ΔbaeJ	BGSC
***B. amyloliquefaciens* FZB42**	ΔbmyA	BGSC
***B. amyloliquefaciens* FZB42**	Δdfn	BGSC

B. amyloliquefaciens FZB42	ΔsrfA	BGSC
P. chlororaphis PCL1606	Wild type	(Cazorla et al., 2006)
P. chlororaphis PCL1606	ΔPCL1606_RS10280 (PvdO)	This study
P. chlororaphis PCL1606	ΔPCL1606_RS14085 (AcsD)	This study
P. chlororaphis PCL1606	ΔPCL1606_RS13600 (PchC)	This study
P. chlororaphis PCL1606	ΔPCL1606_RS05710	This study
P. chlororaphis PCL1606	ΔPCL1606_RS13355	This study
P. chlororaphis PCL1606	ΔPCL1606_RS16180	This study
P. chlororaphis PCL1606	ΔPCL1606_RS15365	This study
P. chlororaphis PCL1606	ΔPCL1606_RS16050	This study
P. chlororaphis PCL1606	ΔPCL1606_RS23355	This study
P. chlororaphis PCL1606	ΔPCL1606_RS08425	This study
P. chlororaphis PCL1606	ΔPCL1606_RS13600 (PchC), ΔPCL1606_RS14085 (AcsD), ΔPCL1606_RS10280 (PvdO)	This study
P. chlororaphis PCL1606	ΔPCL1606_RS13600 (PchC), ΔPCL1606_RS10280 (PvdO)	This study
P. chlororaphis PCL1606	ΔPCL1606_RS10170-RS10180 (DarA-DarB-DarC)	This study
P. chlororaphis PCL1606	ΔPCL1606_RS10170-RS10180 (DarA-DarB-DarC), ΔPCL1606_RS10280 (PvdO)	This study
P. chlororaphis PCL1606	Spontaneous mutant. $fusA^{T680D}$	This study
P. chlororaphis PCL1606	Spontaneous mutant. $fusA^{T680D}$	This study
P. chlororaphis PCL1606	Spontaneous mutant. $fusA^{K366N}$	This study

Table S7. Oligonucleotides used in this study. Related to STAR Methods.

Name	Sequence
T2SS_up_Fwd	agtatagggataacagggtaatctgcaggacccggacatcatcatgatcggc
T2SS_up_Rev	cgataacggttcacaggcgtcccgggtcacacggagga
T2SS_down_Fwd	cccgggacgcctgtgaaccgttatcgctacgaagccgc
T2SS_down_Rev	agaggatccccgggtaccgagctcgcgtgaggatcttgccacgcagggtg
RS05710_up_Fwd	tctgaattcgagctcggtacccgggaccaccggcgggcataagttgtcgtaatt
RS05710_up_Rev	tgcagctgcatgagtcccggaaggggccttcgaccata
RS05710_down_Fwd	ccccttccgggactcatgcagctgcaattcggagccga
RS05710_down_Rev	gcatgcctgcaggtcgactctagagcagctgggtgcggatgaagtgggaaaag
UP_RS14085_fwd	agtatagggataacagggtaatctgcgcgggctgggcctgatgctgggca
UP_RS14085_rev	ggtcgccgcgccaaaaggctccacagtagccatgcgatactggccacg
Down_RS14085_fwd	tgtggagccttttggcgcggcgacctgccgctgctcga
Down_RS14085_rev	agaggatccccgggtaccgagctcgcaactgagtgcgcagacgcaccacccgc
RS13600-up_fwd	agtatagggataacagggtaatctgcgatgaggtgaactcgccgtcattg
RS13600-up_rev	aagggtttttccgggatctcctatttgtagaaataattggctacaaatg
RS13600-DOWN_fwd	aataggagatcccggaaaaacccttccatgaaaacccctgac
RS13600-DOWN_rev	agaggatccccgggtaccgagctcgagaacaggctttcggcgtggggctg
UP_RS10280_Fwd	agtatagggataacagggtaatctgtctgcgcccgcagcacggagctcaa
UP_RS10280_Rev	ggcggtgcgggggggtcgtctcgaaggttggaaaaggtggatcgc
DOWN_RS10280_Fwd	ttcgagacgacccccccgcaccgccccacgccccgga
DOWN_RS10280_Rev	Agaggatccccgggtaccgagctcgggcagcggctacagatgcgagg cgttaacacgacatctgtaggagc
UP_RS10170_Fwd	tctgaattcgagctcggtacccggggtcgaaagagcgggcgccacgatgc
UP_RS10170_Rev	acaggaccggcgaaatacggggaccttttgctttacaaccacaaag
DOWN_RS10170_Fwd	ggtccccgtatttcgccggtcctgtgaagatgcggccg
DOWN_RS10170_Rev	gcatgcctgcaggtcgactctagagctatgtcacctcggtgtgcaccggc
RS15365_UP_Fwd	tctgaattcgagctcggtacccggggagagcgcaatagagcagccaggac
RS15365_UP_Rev	tggcctcccttaatgacgctccataaattaatcctacagtgag
RS15365_Down_Fwd	tatggagcgtcattaagggaggccaggcagcggccgtc
RS15365_Down_Rev	gcatgcctgcaggtcgactctagagtcagcagcctatccatgggagtgaagaccag
bact_RS23355_UP_fwd	tctgaattcgagctcggtacccgggcaggccggtgtgcgtcgtgctgttg
bact_RS23355_UP_rev	cggcttgggtatcgacactcaggcggacggtggattac
bact_RS23355_DOWN_fwd	cgcctgagtgtcgatacccaagccgccttcgccagcgc
bact_RS23355_DOWN_rev	gcatgcctgcaggtcgactctagagggcccatagcgcgaccactggatgag
GlpK_up_Fwd	tctgcagacgcgtcgacgtcatatgtttagatatccacctcggtcaattaaaag
GlpK_up_Rev	aaatggttcgctggatgccgctctcctttttaaatatattc
Km_GlpK_Fwd	gagagcggcatccagcgaaccatttgaggtgataggtaag
Km_GlpK_Rev	cacatttttatccgatacaaattcctcgtaggcgctc
GlpK_down_Fwd	ggaatttgtatcggataaaaatgtggtatactgaaaacaagttaatag
GlpK_down_Rev	tccagcctcgcgtcgggcgatatcgtagagccgtatctgatggctaactg
RS16180_UP_fwd	attcgagctcggtacccgggaagtgccgaggccgaaggaaggaat
RS16180_UP_rev	cgacaacttctgaatcattccaagttctctagttgcgaaaaac
RS16180_DOWN_fwd	gaatgattcagaagttgtcgttggcgtgcagcgcc

RS16180_DOWN_rev	cctgcaggtcgactctagaggttgattccctcttataaccaccgttttctactgg
RS08425_UP_fwd	tctagagtcgacctgcaggcatgcactgatgcgatcggcctcggcccccca
RS08425_UP_rev	tgttgccctgaggcaactctcctgctaccacgctgttttgaatgaac
RS08425_DOWN_fwd	gcaggagagttgcctcagggcaacaccccggctgcccc
RS08425_DOWN_rev	aaaaaagaatatataaggcttttaattccccaccaggtcgaaaccggtcagg
