# Supplementary material for: Chemical interplay and complementary adaptative strategies toggle bacterial antagonism and co-existence
Source: Cell Rep. 2021 Jul 27;36(4):109449. doi: 10.1016/j.celrep.2021.109449 (PMC8333196; doi:10.1016/j.celrep.2021.109449)
Supplement: Document S2. Article plus supplemental information [file mmc11.pdf]

Cell Reports

Article

Chemical interplay and complementary adaptive strategies toggle bacterial antagonism and co-existence

Graphical abstract

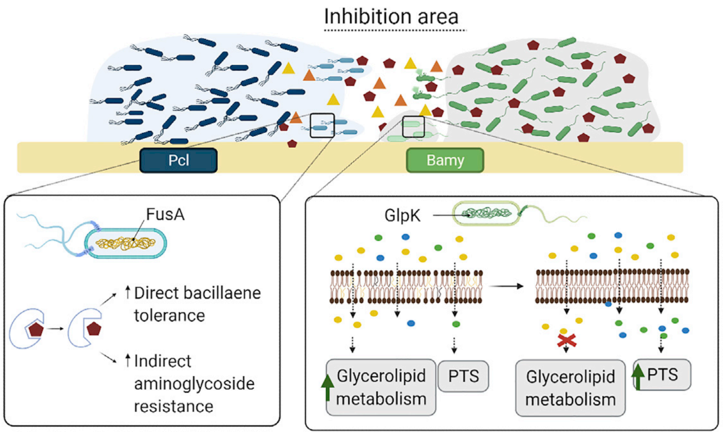

Authors

Carlos Molina-Santiago, David Vela-Corcía, Daniel Petras, ..., Pieter C. Dorrestein, Antonio de Vicente, Diego Romero

Correspondence

diego_romero@uma.es

In brief

Molina-Santiago et al. investigate the spatiotemporal development of the interaction between two plant-beneficial bacterial strains. Bacillaene antibiotic from *Bacillus* inhibits the growth of *Pseudomonas* affecting protein translation. Mutations in genes from central metabolism modify cell membrane composition and rigidity, thereby increasing antibiotic resistance of *Bacillus*.

Highlights

- *Bacillus* and *Pseudomonas* interaction ranges from antagonism to co-existence
- Bacillaene from *Bacillus* is a bacteriostatic that targets FusA of *Pseudomonas*
- GlpK mutations in *Bacillus* confer unspecific antimicrobial resistance

Molina-Santiago et al., 2021, Cell Reports *36*, 109449
July 27, 2021 © 2021 The Author(s).
https://doi.org/10.1016/j.celrep.2021.109449

CellPress

Cell Reports

CellPress
OPEN ACCESS

Article

Chemical interplay and complementary adaptative strategies toggle bacterial antagonism and co-existence

Carlos Molina-Santiago,[1] David Vela-Corcía,[1] Daniel Petras,[2,3] Luis Díaz-Martínez,[1] Alicia Isabel Pérez-Lorente,[1] Sara Sopeña-Torres,[1] John Pearson,[4] Andrés Mauricio Caraballo-Rodríguez,[3] Pieter C. Dorrestein,[3] Antonio de Vicente,[1] and Diego Romero[1,5,*]

[1]Instituto de Hortofruticultura Subtropical y Mediterránea "La Mayora," Universidad de Málaga-Consejo Superior de Investigaciones Científicas (IHSM-UMA-CSIC), Departamento de Microbiología, Universidad de Málaga, Bulevar Louis Pasteur 31 (Campus Universitario de Teatinos), 29071 Málaga, Spain
[2]University of California San Diego, Scripps Institution of Oceanography, La Jolla, CA, USA
[3]University of California San Diego, Collaborative Mass Spectrometry Innovation Center, La Jolla, CA, USA
[4]Nano-imaging Unit, Andalusian Centre for Nanomedicine and Biotechnology, BIONAND, Málaga, Spain
[5]Lead contact
*Correspondence: diego_romero@uma.es
https://doi.org/10.1016/j.celrep.2021.109449

SUMMARY

Bacterial communities are in a continuous adaptive and evolutionary race for survival. In this work we expand our knowledge on the chemical interplay and specific mutations that modulate the transition from antagonism to co-existence between two plant-beneficial bacteria, *Pseudomonas chlororaphis* PCL1606 and *Bacillus amyloliquefaciens* FZB42. We reveal that the bacteriostatic activity of bacillaene produced by *Bacillus* relies on an interaction with the protein elongation factor FusA of *P. chlororaphis* and how mutations in this protein lead to tolerance to bacillaene and other protein translation inhibitors. Additionally, we describe how the unspecific tolerance of *B. amyloliquefaciens* to antimicrobials associated with mutations in the glycerol kinase GlpK is provoked by a decrease of *Bacillus* cell membrane permeability, among other pleiotropic responses. We conclude that nutrient specialization and mutations in basic biological functions are bacterial adaptive dynamics that lead to the coexistence of two primary competitive bacterial species rather than their mutual eradication.

INTRODUCTION

Microbes living in multispecies communities are continuously interacting and competing for scarce resources such as nutrients and space, which in the end are key determinants of the evolution and the success of a community (Granato et al., 2019). Thus, an understanding of how bacterial species interact, communicate, and evolve to coexist or to defeat competitors is a major interest in microbial ecology (Niehaus et al., 2019). Specifically, the plant niche often represents a competitive environment where microbes are in a continuous fight for nutrients, either secreted by the plant or found in the soil (Bauer et al., 2018; Kuzyakov and Xu, 2013; Vieira et al., 2020). Bacterial competition for nutrients and space can be ecologically and evolutionary encompassed in the game theory introduced by Smith and Price (1973). Most recently, some studies have focused on analyzing bacterial fight, as well as the relevance of resources, space, cell density, reciprocation, and provocation, in bacterial competition and co-existence (Cornforth and Foster, 2013; Ghoul and Mitri, 2016; Granato et al., 2019). Competition is favored when coexisting strains require similar resources, or cells are mixed where nutrients and secretions are shared (Ghoul and Mitri, 2016). In addition, a competition sensing hypothesis proposes the ability of bacterial cells to detect and respond to competition due to physiological stress responses and sensing mechanisms (Cornforth and Foster, 2013; LeRoux et al., 2015). In these scenarios, the activation of specific metabolic pathways and the production and secretion of signaling compounds, siderophores, antibiotics, and quorum-sensing molecules are bacterial factors that mediate interspecies and intraspecies interactions as well as inter-kingdom communication (Benoit et al., 2015; Parsek and Greenberg, 2005; Puri et al., 2018; Stempler et al., 2017).

Bacillus and *Pseudomonas* are two of the most predominant bacterial genera found in plant microbiomes (Lozano et al., 2019; Wei et al., 2019), and their beneficial effects on plants in the fight against fungal and bacterial pathogens via direct antagonism or indirectly via the activation of plant defense mechanisms (ISR) have been extensively studied (Berlanga-Clavero et al., 2020; Kloepper et al., 2004). However, how *Pseudomonas* and *Bacillus* strains coexist has only been partially described. Separate studies have revealed (1) the relevance of sporulation and biofilm formation in the protection of *Bacillus* against *Pseudomonas* competition, and (2) molecules of *Pseudomonas* that

This is an open access article under the CC BY license (http://creativecommons.org/licenses/by/4.0/).

inhibit cell differentiation of *Bacillus* and the relevance of the type VI secretion system (T6SS) as an offensive tool in close cell-to-cell contact (Cámara-Almirón et al., 2020; Molina-Santiago et al., 2019). Despite the major role that these processes play in bacterial interactions, it is well known that bacteria produce a vast arsenal of toxins and secondary metabolites that play critical roles in the modulation of antagonistic interactions (Berlanga-Clavero et al., 2020; Bernal et al., 2018; Ghequire and De Mot, 2015).

Bacillus amyloliquefaciens FZB42 (Bamy) is a Gram-positive soil bacterium with outstanding potential for the production of non-ribosomal secondary metabolites (Chen et al., 2009b), e.g., fengycins, surfactins, bacillaene, bacillomycin D, bacillibactin, difficidin, bacilysin, and other unknown compounds, that participate in diverse biological and communicative processes (Chen et al., 2009a). In fact, it has been proposed that 8.5% of the Bamy genome is devoted to non-ribosomal biosynthesis of secondary metabolites, highlighting the relevance of these molecules to the lifestyle of this bacterium (Chen et al., 2009b). Among pseudomonads, *Pseudomonas chlororaphis* PCL1606 (Pcl) is a well-known bacterium that was isolated from the rhizosphere of avocado trees (Cazorla et al., 2006) and has shown antifungal and antimicrobial activities thanks to its production of several small molecules, including pyrrolnitrins, hydrogen cyanide, and 2-hexyl-propyl-resorcinol (HPR) (Calderón et al., 2015). In addition, genomic sequence analysis has determined that Pcl produces siderophores such as pyochelin, pyoverdine, and achromobactin (Arrebola et al., 2019; Calderón et al., 2015).

In this work we intended to decipher the mechanisms underlying bacterial interactions between plant-beneficial bacteria and to determine the genetic modifications that help *Bacillus* and *Pseudomonas* strains to ecologically evolve from an antagonistic interaction to the co-existence between species in the rhizosphere. We have found that, in the short-term, Pcl inhibits the growth of Bamy via the combined effects of molecules regulated by the two-component system GacA-GacS while the production of the bacteriostatic compound bacillaene protects Bamy from Pcl advance. Mutations in the specific target of the antimicrobial compounds or enzymes produced by Bamy and Pcl, with the consequent pleiotropic effect, arise as the most favorable strategies leading to the co-existence of both bacterial species. Our results serve to decipher the intricacies of bacterial communication and the adaptive response to antimicrobial-mediated inhibition that support stable mixed communities.

RESULTS

The interaction between Pcl and Bamy progresses from antagonism to coexistence

Pairwise interaction experiments are routinely used as the starting point for exploring the type of ecological interactions occurring between microorganisms, from antagonism to cooperation by different ways of bacterial communication. In a previous study we found that different growth media inflicted variations in the resulting interaction between *Bacillus subtilis* and Pcl (Molina-Santiago et al., 2019). The two strains come into close contact in lysogeny broth (LB) medium, while in King's B medium, which promotes the production of secondary metabolites by *Pseudomonas* strains (Rieusset et al., 2020), the two colonies were physically separated by a typical inhibition halo (Figure S1A). This result was reproducible in the interaction between Pcl with Bamy, a closely related species to *B. subtilis* (Figure 1A, top; Figure S1B). In addition, pairwise interactions of Pcl and Bamy in minimal medium M9 supplemented with glycerol as sole carbon source showed a similar behavior to that observed in a rich medium such as King's B (Figure S1C), although with a lower inhibitory effect. Pcl and Bamy are strong producers of a broad range of secondary metabolites, and they are natural inhabitants of the rhizosphere where they contribute to the health and yield of plants in different ways. For these reasons, we selected these strains and King's B medium to characterize the mechanism of the interspecies chemical interplay.

Time-course analysis of the Pcl-Bamy interaction in King's B medium suggested mutual antagonism (Figure 1A, bottom), an idea that we explored at the cellular level via time-lapse microscopy analysis of their interaction (Figures 1B and 1C; Video S1). At the initial stages of colony growth, the expansion rate of Bamy was 5-fold faster than that of Pcl (Figure 1C). After 9 h of interaction, Bamy growth was arrested, and the leading Pcl-proximal edge showed initial signs of cell death (Figures 1B and 1D) while Pcl maintained the same displacement rate. After 30 h, Pcl arrived at the "death zone" of the Bamy colony and progressively reduced the expansion rate, concomitant with a second wave of cell death in the inner areas of the Bamy colony (which was not in physical contact with Pcl) (Figure 1C). The interaction stabilized after 60 h, and no further inhibition of Bamy colony growth or Pcl expansion was observed. At later time points (90–120 h), small colonies of Bamy grew in the inhibition area (macroscopic image in Figure 1A [96 h]), and spontaneous clones emerged from the leading edge of the original Pcl colony, which were capable of reaching the inhibition zone (Figure 1B [126 h]). These results were consistent with our initial hypothesis of mutual exclusion between the two species. In addition, colony behavior at the cellular level and the particular emergence of spontaneous mutants in each strain collectively suggested that Pcl might secrete bactericidal molecules active against Bamy, while bacteriostatic molecules from Bamy seemed to be more relevant in this interspecies interaction.

Pcl uses the two-component system GacA-GacS to deploy its antimicrobial activity

The previous findings furthered the study of the interaction at two different stages: short-term (24–48 h), to delineate the chemical interplay mediating the antagonism between the two strains; and long-term (96–144 h), to define the genetic changes that lead to their adaptation and coexistence in this adverse chemical environment. Thus, we first analyzed gene expression changes at 24 h to determine whether biofilm-related pathways were differentially expressed and to seek putative antimicrobials potentially induced during the interaction (Figures 2A and 2B; Figure S2; Tables S1 and S2). The number of differentially expressed genes in Pcl (304 induced and 59 repressed, $p < 0.05$) was larger than that in Bamy (73 induced and 58 repressed, $p < 0.05$). Kyoto Encyclopedia of Genes and Genomes (KEGG) pathway and Gene

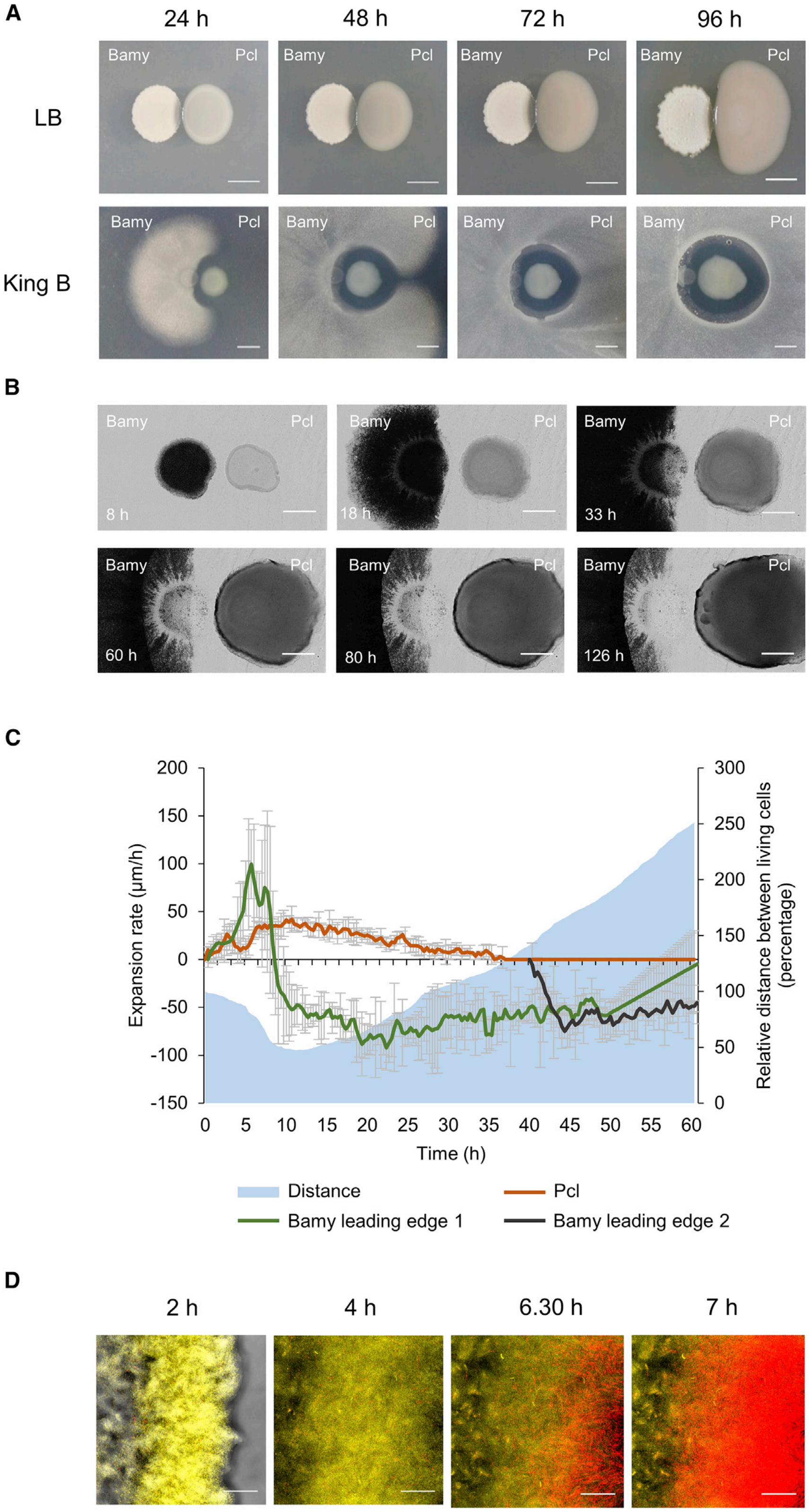

Figure 1. Pcl and Bamy mutually exclude each other in King's B medium

(A) Pairwise interaction time lapses between Bamy (left) and Pcl (right) on LB (top) and King's B (bottom) media at 24, 48, 72, and 96 h. Scale bars, 5 mm.

(B) Time-lapse microscopy of the pairwise interaction between Pcl and Bamy during 126 h. Scale bars, 2 mm.

(C) Expansion rates of the Bamy and Pcl leading edges and distance between both strains during the interaction. The orange line represents the Pcl leading edge, and the green and black lines represent Bamy leading edges 1 and 2, respectively. Error bars represents SD. n = 3. The blue area represents the distance between the two populations during the entire interaction.

(D) Confocal laser scanning microscopy (CLSM) time-course experiment of the interaction area using propidium iodide (PI) to identify cell death. Bamy is fluorescently labeled with yellow fluorescent protein (YFP). Scale bars, 40 µm.

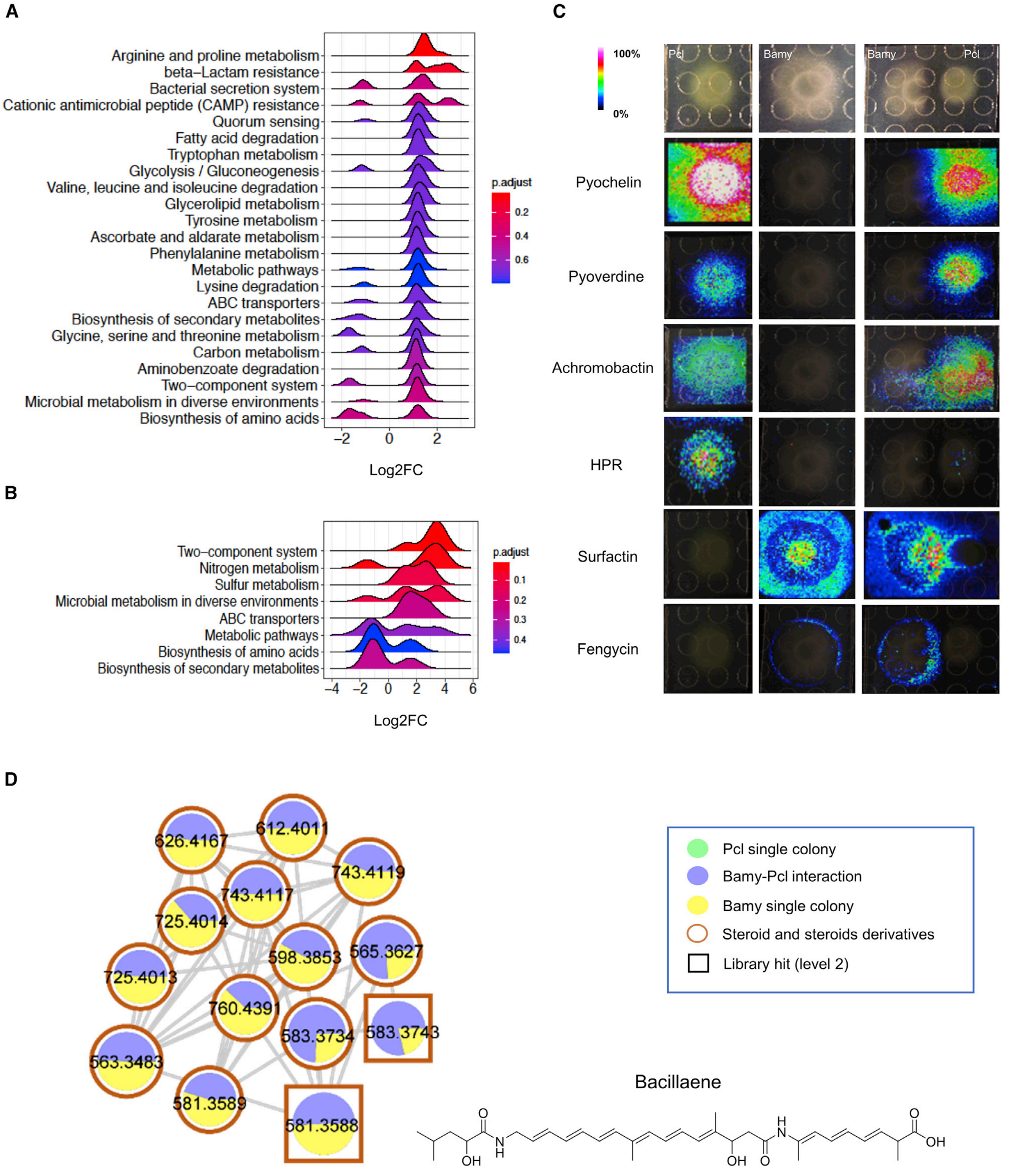

Figure 2. Production and spatial distribution of putative antimicrobials by Pcl and Bamy

(A and B) Transcriptional analysis of overexpressed KEGG pathways in (A) Pcl and (B) Bamy after interaction for 24 h.

(C) MALDI-MSI heatmaps showing the spatial distribution of siderophores (pyoverdine, pyochelin, and achromobactin) and other secondary metabolites (HPR, surfactin, and fengycin) produced by Pcl and Bamy growing alone or in interaction.

(D) Molecular family of bacillaene detected from Bamy grown as a single colony or in the interaction with Pcl. Results were obtained in mass spectrometry analysis using LC-MS/MS and feature-based molecular networking. Each metabolite is represented by a circle, and they are connected according to the mass

(legend continued on next page)

Ontology (GO) term analyses showed that pathways related to central metabolism and the cell membrane were mostly altered in Pcl (Figure 2A; Figures S2A and S2B). Specifically, we found induction of (1) secondary metabolites, e.g., achromobactin or other gene clusters predicted to participate in the production of secondary metabolites according to AntiSmash (Blin et al., 2019); and (2) the type II secretion system (T2SS) and many efflux pumps. Interestingly and contrary to the interaction between *B. subtilis* and Pcl in LB medium (Molina-Santiago et al., 2019), the expression of the T6SS was downregulated, most likely due to the absence of close cell-to-cell contact (Figure 1A). Other downregulated genes were dedicated to the synthesis of the siderophore pyochelin and the antimicrobial pyrrolnitrin (Figure 2C; Table S1). In Bamy, extracellular matrix- and sporulation-related genes were not differentially expressed. The main pathways overexpressed were related to (1) sulfur and nitrogen metabolism and the phosphotransferase system (PTS) (Figure 2B), and (2) synthesis of the antimicrobials bacillaene and difficidin (Table S2). Histidine metabolism (Figure 2B) and many of the genes related to membrane proteins and the glycerol uptake system were mostly repressed (Table S2; Figures S2C and S2D).

In light of these results, we analyzed the presence and distribution of metabolites produced by each strain using matrix-assisted laser desorption ionization mass spectrometry imaging (MALDI-MSI) and liquid chromatography-tandem mass spectrometry (LC-MS/MS) non-targeted metabolomics along with the detection of putative inhibitory molecules (Figures 2C and 2D; Figure S3; Table S3). Although differentially expressed at the transcriptional level (Table S1), the siderophores achromobactin, pyochelin, and pyoverdine were distributed around the Pcl colony in the inhibition area and in the inner colony during the interaction (Figures 2C and 2D; Figure S3). The fractionation analysis of Pcl cell-free supernatants confirmed that fractions containing pyochelin methyl ester (*m/z* 339.08), uniquely detected in the interaction (Figure S4A), HPR (*m/z* 237.18), or two additional unidentified molecules, were the most potent inhibitors of Bamy growth (Table S3). We were, however, unable to detect achromobactin (*m/z* 592.16) in any of the fractions tested. Unexpectedly, null single pyochelin mutant or even double and triple mutants in the remaining siderophores (achromobactin and pyoverdin) still antagonized Bamy at comparable levels to wild-type (WT) Pcl (Figure S4A). Diverse lines of evidence also discounted the involvement of HPR in the antagonistic interaction, including (1) downregulation of gene expression during the interaction, (2) lack of diffusion of the molecule from the Pcl colony as revealed by MALDI-MSI (Figure 2C), and (3) antagonistic activity of a null HPR mutant comparable to the WT (Figure S4A). Mutants in the T2SS, the most overexpressed efflux pump, and the other non-ribosomal peptide synthetase (NRPS) clusters predicted by AntiSmash still retained comparable antimicrobial activity to WT Pcl (Figures S4A and S4B). Untargeted metabolomics and feature molecular networking (Nothias et al., 2020) using the GNPS platform (Wang et al., 2016) and MALDI-MSI experiments showed two additional molecular families produced exclusively by Pcl or primarily during the interaction with a distribution pattern consistent with putative involvement in the antagonism (Figures S5A and S5B). *In silico* analysis with MolNetEnhancer and network annotation propagation (NAP) (da Silva et al., 2018; Djoumbou Feunang et al., 2016; Ernst et al., 2019) led us to classify these molecules as glycerophosphoethanolamines and benzene derivatives at the subclass level (Djoumbou Feunang et al., 2016). The complementary use of SIRIUS/ZODIAC (Dührkop et al., 2021), CSI:FingerID (Dührkop et al., 2019), and CANOPUS (Dührkop et al., 2021) predicted molecular formulas, compound class, and putative chemical structure based on *in silico* MS/MS fragmentation trees. The results obtained for both molecular families were consistent with the MolNetEnhancer results and confirmed the presence of both phosphoethanolamines and benzene derivatives (Figures S5A and S5B); however, the lack of more information on these molecules precludes us from reaching more definitive conclusions.

The combination of metabolomics and functional genetics suggested that Pcl antagonism is multifactorial rather than based on single and known antimicrobials. Thus, we built a library of transposon random mutants to alternatively identify putative candidates involved in this inhibitory activity. As previously stated by Turner et al. (2014) and Valli et al. (2020), we found no correlation between transcriptomic results and transposon library screening. Clones with no antimicrobial activity shared mutations in the gene RS_08425, which encodes GacS, a member of the GacA-GacS two-component system. Knockout mutants in other candidate genes did inhibit antagonism. Pairwise interactions and time-lapse microscopy analyses of *ΔGacS* and Bamy showed no inhibition between the two strains and even overgrowth of Bamy on the leading edge of a *ΔGacS* colony (Figures S5C and S5D; Video S2). Overall, we concluded that Pcl antagonism is more complex than anticipated and most likely involves several secondary metabolites and other metabolic derivatives, all under control of the environment-sensing GacA-GacS two-component system.

The inhibitory actions of Bamy rely on the bacteriostatic compound bacillaene

Transcriptomic analysis revealed overexpression of bacillaene and difficidin by Bamy as a consequence of its interaction with Pcl (Table S2), and non-targeted metabolomics confirmed bacillaene overproduction. MALDI-MSI analysis added surfactin and fengycin to the group of putative antimicrobials of Bamy that might mediate its antagonistic interaction with Pcl (Figure S2). Fengycin and bacillomycin are well-known antifungal compounds with little or null antibacterial activity, and, accordingly, pairwise interactions between strains of single mutant for either of these molecules (*ΔfenA* or *ΔbmyA*) and Pcl provided comparable antagonistic results to those of WT Bamy (Figures 3A and 3B; Figure S6A). A surfactin mutant (*ΔsrfA*) showed reduced colony expansion (an expected finding) but antagonized Pcl comparably to WT Bamy. The size of the inhibition area was, however, mildly reduced in the interaction with a difficidin mutant

fragmentation patterns. The chemical structures of the annotated features are based on spectral matches in GNPS libraries representative of specific molecular families. Border colors indicate ClassyFire classification. The sizes of the compounds are directly related to their abundance in the metabolome. Squares indicate a library hit level 2 through GNPS, and circles indicate unknown compounds based on GNPS searches.

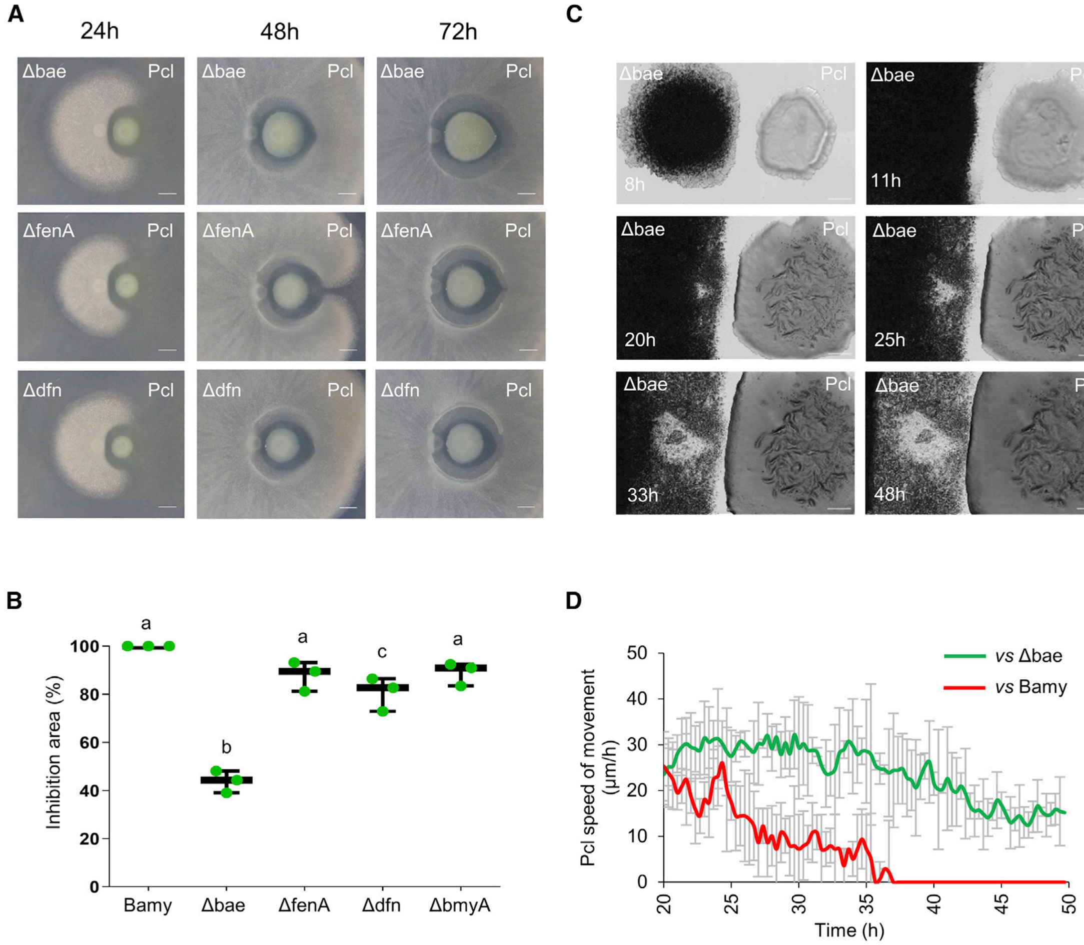

Figure 3. Bacillaene is a bacteriostatic compound produced by Bamy that arrests Pcl growth

(A) Time-course pairwise interactions between Pcl and Bamy mutants in secondary metabolites bacillaene (*Δbae*), fengycin (*ΔfenA*), and difficidin (*Δdfn*) on King's B medium at 24, 48, and 72 h. Scale bars, 5 mm.

(B) Measurement, in percentage, of the inhibition area compared with the WT interaction between Pcl and Bamy mutants at 72 h. Mean values of three biological replicates are shown, with error bars representing SEM. The same letters indicate differences that were not significant (a = 0.05), using a one-way ANOVA followed by a Dunnett's test, n = 3.

(C) Time-lapse microscopy of the pairwise interaction between Pcl and Δbae during 48 h. Scale bars, 2 mm.

(D) Expansion rates of the Pcl leading edges in the interaction with WT Bamy (red line) and *Δbae* (green line) from 20 to 50 h. Error bars represents SD. n = 3.

(*Δdfn*) (Figure 3A) and strongly diminished (60% compared to the interaction with WT Bamy) in the absence of bacillaene (*Δbae*) (Figure 3B). Two additional findings supported the inhibitory contribution of bacillaene to the interaction: (1) the disappearance of most of the inhibition halo after 72 h; and (2) the larger size of the Pcl colony in comparison with those of the other interactions (Figure 3A). Time-lapse microscopy experiments confirmed that, indeed, the larger size of the Pcl colony was due to growth beyond the area initially colonized by the *Δbae* colony (Figure 3C; Video S3). A comparison of the speed of movement clearly showed that Pcl expanded constantly at 25–30 μm/h in the interaction with *Δbae* (Figure 3C), but that it completely ceased after 35 h of interaction with WT Bamy. These results provide support for a protective role of bacillaene produced by Bamy against the advance of Pcl; furthermore, the lack of massive cell death at the front line of the Pcl colony also led us to confirm that bacillaene exerts a bacteriostatic effect (Patel et al., 1995).

Mutations in FusA overcome bacillaene-mediated antagonism

As revealed earlier via time-lapse microscopy analysis, Bamy colonies spontaneously emerged in the inhibition zone, and clones grew from the Pcl colony when the interaction stabilized after 90 h (Figure 1B; Figure 4A; Video S1). To elucidate the genomic changes driving this microbial adaptation, we sequenced the genomes of three spontaneous Pcl clones and seven spontaneous Bamy clones. Comparison of the Pcl clones showed a variety of point mutations at different loci, and recurring changes were found in the locus PCL1606_RS29735, which encodes translation elongation factor G (FusA) (Table 1). No inhibition halos were observed

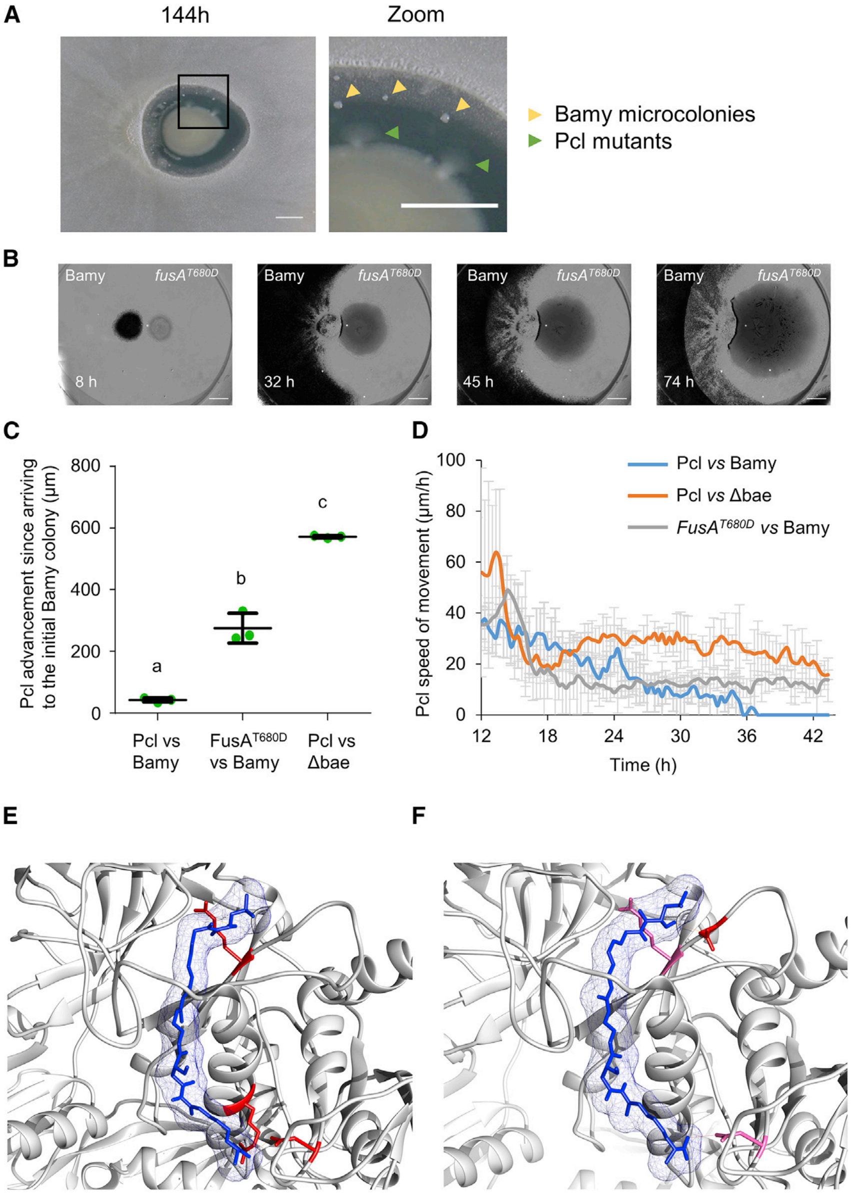

Figure 4. Mutations in Pcl FusA confer bacillaene resistance

(A) Pairwise interaction between Pcl and Bamy after interaction for 144 h. Magnification of the selected area permits visualization of Bamy microcolonies (yellow triangles) and Pcl subzones (green triangles) growing in the inhibition area. Scale bars, 5 mm.

(B) Time-lapse microscopy of the pairwise interaction between FusA^{T680D} and Bamy after 74 h. Scale bars, 2 mm.

(C) Measurement of the Pcl colony movement within 24 h of their arrival at the initial position of a Bamy colony. Mean values of three biological replicates are shown, with error bars representing SEM. The same letters indicate differences that were not significant (a = 0.05), using a one-way ANOVA followed by a Dunnett's test. n = 3.

(D) Expansion rates of the WT Pcl leading edges during interaction with WT Bamy (blue line), *Δbae* (orange line), and the FusA^{T680D} leading edge expansion rate during interaction with Bamy from 12 to 43 h. Error bars represents SD. n = 3.

(E) Molecular docking between bacillaene and FusA reveals a putative binding site formed by Arg29, Arg89, and Asn272 (highlighted in red).

(F) Molecular docking between bacillaene and FusA^{T680D} reveals changes in the amino acids involved in the binding site (Asn81, Arg83, and Asn272). Residues highlighted in pink are shared with the WT model while residues in red are the changing residues.

at late time points during pairwise interactions between these Pcl clones and WT Bamy, a pattern similar to that of the interaction between WT Pcl and *Δbae* (Figure S6B). These results show that the speed of movement of Pcl clones, recorded via time-lapse microscopy experiments (Figure 4B; Video S4), was constant during the interaction and similar to that during the interaction between WT Pcl and *Δbae*, although forward movement decelerated relative to that observed in the complete absence of bacillaene (Figures 4C and 4D).

Previous studies have demonstrated that *Staphylococcus aureus* FusA is the target of the antibiotic fusidic acid (Besier et al., 2003; Chen et al., 2010). In addition, FusA has also been revealed to be involved in tolerance of the aminoglycoside antimicrobials gentamicin, amikacin, and tobramycin by *P. aeruginosa* (Bolard et al., 2018). FusA is highly conserved, and its three-dimensional structure has been elucidated, enabling *in silico* molecular docking studies of the WT protein or mutated proteins in the presence and absence of bacillaene. Molecular modeling of WT FusA and FusA^{T680D} showed slight structural and conformational changes in the most probable bacillaene binding pocket (Video S5). Molecular docking of bacillaene and WT FusA resulted in 48 identifiable clusters in eight different sites in the protein. The top-score cluster exhibited higher full fitness and lower free energy values of −5,458.2 and −7.8 kcal/mol, respectively, compared with those of other potential binding sites. In its interaction with WT FusA, bacillaene formed one hydrogen bond with the secondary amine of Arg29 (2.97 Å), one with the secondary amine of Arg89 (2.1 Å), and one with the primary amine of Asn272 (3.101Å), suggesting a favorable binding site (Figures 4E and 4F). However, molecular docking of bacillaene and FusA^{T680D} resulted in the identification of nine clusters in two different sites in the protein. The top-score cluster exhibited higher full fitness and free energy values of −2,187.4 and −7.6 kcal/mol, respectively, compared with those of WT FusA. In this interaction, three hydrogen bonds were predicted: one hydrogen bond with the hydroxyl group of Asn81 (2.17 Å), one with the secondary amine of Arg83 (3.10 Å), and one with the primary

Table 1. Alleles of *fusA* and *glpK* identified in Pcl and Bamy spontaneous mutants, respectively

Allele	Nucleotide change	Amino acid change	Resistance to Bamy	Resistance to Pcl
fusA^{T680D}	g.2038A>G	p.T680D	+	
fusA^{T680D}	g.2038A>G	p.T680D	+	
fusA^{K366N}	g.1098G>T	p.K366N	+	
glpK$^{F38S, W52stop, G254A}$	g.T113C	p.F38S		+
	g.155G>A	p.W52stop		
	g.761G>A	p.G254A		
glpK$^{G147R, Q421R}$	g.409G>A	p.G147R		+
	g.1262A>G	p.Q421R		
glpK^{W355R}	g.1063T>C	p.W355R		+
glpK^{S166L}	g.497C>T	p.S166L		+
glpK^{+}	g.1260C>T	N/A		–
glpK$^{G162R, W485stop}$	g.484G>A	p.G162R		+
	g.1455G>A	p.W485stop		
glpK$^{Q246-, G265S}$	g.736C>T	p.Q246-		+
	g.793G>A	p.G265S		

amine of Asn272 (2.94 Å) (Figures 4E and 4F), suggesting a lower affinity for bacillaene with the mutant FusA and, thus, reduced inhibitory activity.

Based on our model, we hypothesized that mutations in FusA would also decrease its affinity for fusidic acid, thus promoting higher tolerance to this molecule (Figures S6C and S6D). The top-score cluster obtained via molecular docking of WT FusA with fusidic acid revealed full fitness and free energy values of −3,257.2 and −8.7 kcal/mol, respectively, with five hydrogen bonds predicted: one with the hydroxyl group of Glu98 (2.21 Å), one with the hydroxyl group of Thr89 (2.18 Å), two with the primary and secondary amines of Arg101 (3.027 and 3.13 Å), and one with the hydroxyl group of Thr402 (2.98 Å), suggesting a favorable binding site. The same analysis with the FusA^{T680D} model exhibited a top-score cluster with full fitness and free energy values of −2,180.025 and −6.98 kcal/mol, respectively. Three hydrogen bonds were predicted: one with the secondary amine of Arg101 (2.54 Å) and two with Thr402 (one with the hydroxyl group [2.51 Å] and one with its secondary amine [2.33 Å]). Molecular modeling of fusidic acid and FusA was experimentally confirmed using minimal inhibitory concentration (MIC) experiments demonstrating that the FusA^{T680D} strain is four times more resistant to fusidic acid than is Pcl (Table S4). These results are consistent with those of bacillaene, providing strong evidence of its mechanism of action. Interestingly, we also noticed that mutations in FusA provided protection against other antibiotics that target the translation machinery. MIC experiments using the aminoglycoside antibiotics kanamycin and gentamicin, which target ribosomal subunits, confirmed that these single amino acid mutations in FusA resulted in higher levels of resistance, i.e., 2- and 10-fold, respectively (Table S4).

Based on these results, we conclude that the antimicrobial activity of bacillaene stems from its inhibition of protein translation via direct interaction with FusA and that point mutations in the protein, which preserve its function, provide resistance to bacillaene and other unrelated protein translation inhibitors.

GlpK promotes pleiotropic cellular changes and unspecific antibiotic resistance

Genome sequence analysis and comparison from seven Bamy isolates showed only a few mutations at multiple loci (Figure S6E). Mutations in multiple residues were only found in *glpK* (Table 1). Two of the clones showed mutations that result in premature stop codons; one mutation was silent (no amino acid substitution), and the remaining mutations accumulated 1- or 2-nt changes that resulted in amino acid substitutions. Phenotypically, these clones expanded less than the WT Bamy in King's B medium and in glycerol-supplemented LB medium (Figures S6F, S6G, S7A, and S7B), demonstrating the relevance and direct involvement of glycerol metabolism in the expansion of these colonies. In interactions with Pcl, the *glpK* mutants reached the Pcl colony, although initial signs of cell death were again observed at the Pcl-proximal colony edge (Figure 5A, top). To confirm that these findings were associated with the loss of GlpK function, we constructed a *glpK* null strain via deletion of *glpK*, and this strain phenotypically mirrored the isolated spontaneous clones (Figure 5A, bottom). Examination of the interaction between GlpK^{S166L} and WT Pcl at the cellular level via time-lapse microscopy experiments demonstrated partial inhibition of *Bacillus* growth at the Pcl-proximal leading edge of the colony (Figure 5B; Video S6). However, notable differences in the interaction between WT Bamy and Pcl included (1) lack of massive cell death in the leading edge of GlpK^{S166L}, which permitted physical contact between the two colonies (Figures 5B and 5D), and (2) absence of regression of a secondary leading edge of the GlpK^{S166L} strain after 40 h of interaction (Figure S8A).

We next wanted to determine the genetic basis of the phenotypic changes in the *glpK* mutants and the highest tolerance to Pcl antagonism. Comparative transcriptomic analysis of the GlpK^{S166L} mutant strain and WT Bamy revealed a vast number of differentially expressed genes related to central metabolism and sugar uptake (Figure 5E; Table S5). As expected from *glpK*

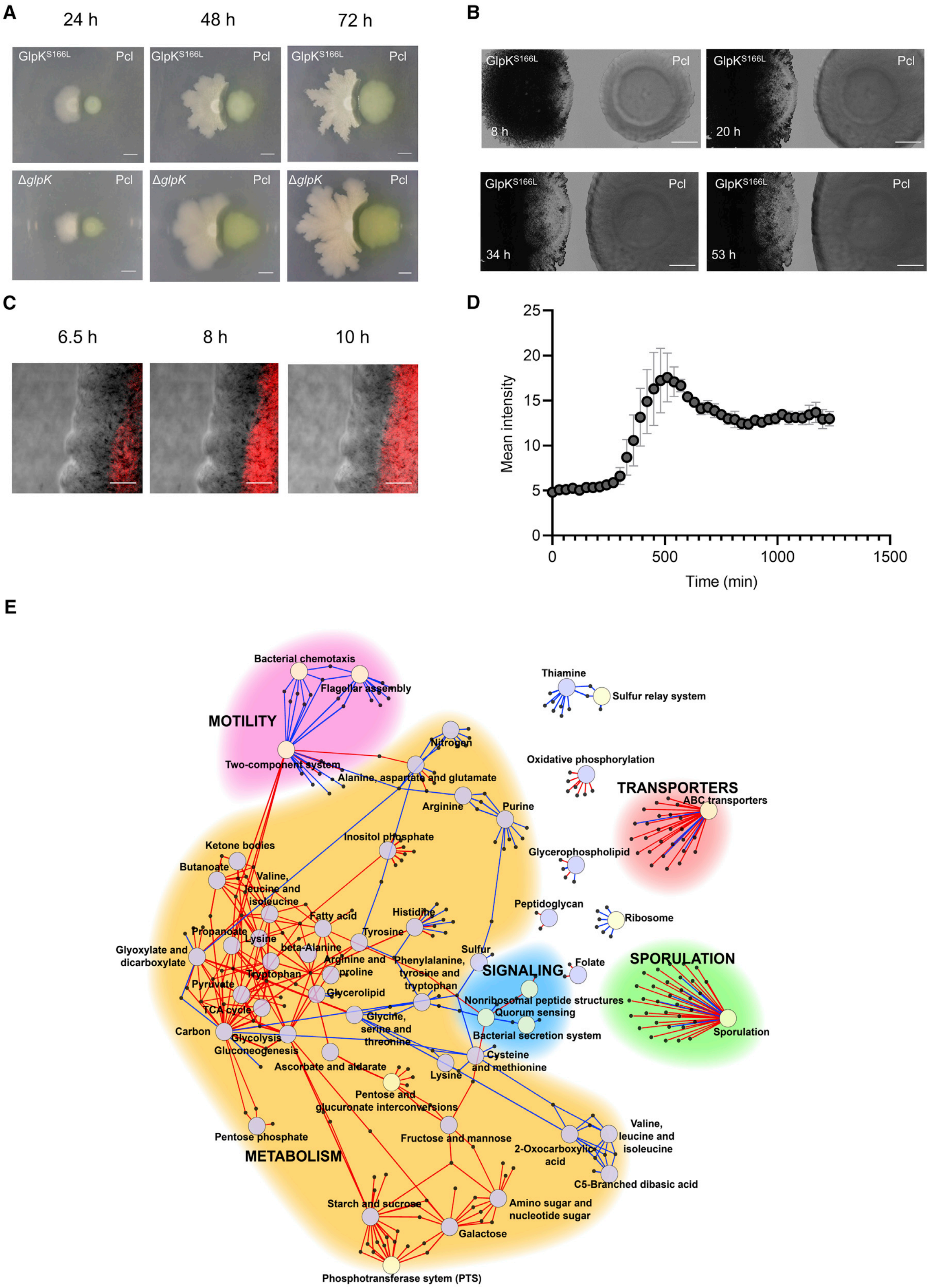

(legend on next page)

mutants, which are incapable of metabolizing glycerol, the main carbon source in King's B medium, we found overexpression of PTS and the pathways involved in the uptake of galactose, starch and sucrose, fructose, or mannose (Figure S8B). Additionally, the tricarboxylic acid (TCA) cycle, pentose phosphate pathway, and fatty acid metabolism were also induced, while the expression levels of genes involved in amino acid biosynthesis and metabolism were only mildly activated or repressed (Figure 5D; Table S5). Reduced swarming motility (Figure S8C) reflected repression of motility- and quorum sensing-related genes. The expression levels of genes associated to secretion systems and sporulation were induced compared with their levels in WT Bamy.

Complementary metabolomic analysis revealed a pronounced reduction of surfactin production, which is likely associated with the reduced swarming motility of *glpK* mutants, slight changes in bacillaene production, and increased fengycin production (Figure S8D). Corroborating the transcriptomic data, metabolomics analysis also indicated important changes in the metabolic pathways involved in fatty acid metabolism, specific changes in the composition of phosphoethanolamines of Bamy cells (Figures 5E and 6A; Table S5), and the absence of or decrease in the levels of glycerophosphoethanolamines and benzene derivatives (Figure S9A), which are candidate inhibitory compounds produced by Pcl.

We predicted that changes in the overall fatty acid content of *glpK* mutant cells could alter the functionality of cell membrane lipids, possibly underlying the higher protection against Pcl chemical aggression. Confocal microscopy analysis of cultures treated with the membrane dye DilC12 revealed the presence of highly fluorescent foci, which correspond to regions with increased fluidity, distributed along the membrane of WT Bamy cells. However, the membranes of GlpK^{S166L} cells stained uniformly with no signs of patches of dye accumulation (Figure 6B, left; Figure S9B), which indicated higher rigidity and, thus, reduced permeability. We also observed significant decreases in membrane potential and the respiratory activity of GlpK^{S166L} cells in tetramethylrhodamine methyl ester (TMRM) and 5-cyano-2,3-ditolyl-tetrazolium chloride (CTC) experiments, respectively (Figure 6B, center and right; Figures S9C–S9E). These results reveal that GlpK confers an unexplained tolerance to antibacterial compounds produced by Pcl; thus, mutations in this gene are selected as the most favorable evolutionary trait in the face of diverse chemical aggression (Bryan and Kwan, 1983; Taber et al., 1987).

DISCUSSION

Bacterial interactions are normally considered from an antagonistic perspective, and ongoing efforts are dedicated to identifying the specific inhibitory metabolites. However, bacterial interactions are diverse, and the involvement of specific mechanisms largely depend on cell-to-cell distance and the environment (Figure 6C). In close cell-to-cell interactions, injection structures, e.g., the T6SS (Lin et al., 2019; Lopez et al., 2020; Molina-Santiago et al., 2019; Perault et al., 2020), ensure the efficient delivery of the inhibitory compounds, while in long-distance interactions, diffusible molecules are more important (Deveau et al., 2016; Tyc et al., 2017). Inhibitory molecules are not always produced or concentrated at lethal levels, and alternative modes of action that induce changes in gene expression patterns or in modulation of the behavior of the competitor have emerged (Andersson and Hughes, 2014; Molina-Santiago et al., 2015). Thus, interspecies interactions are not classified as beneficial or detrimental based on specific time frames; however, these interactions represent a fascinating scenario where competitors attempt to adapt and survive in the presence of other species in the same ecological niche (Turcotte et al., 2012). In this work, we provide substantial insight into the evolution of interspecies interactions by studying the mechanism of the chemical interplay between Pcl and Bamy, two plant-beneficial bacteria (Berlanga-Clavero et al., 2020; Calderón et al., 2015; Chen et al., 2009a; Niehaus et al., 2019). Understanding of this interaction is also important from a biotechnological perspective, given that their beneficial effects can be potentiated or inhibited depending on how the strains interact in the short term and adapt and evolve to ensure subsistence and/or coexistence in the long term.

In a previous study we demonstrated the relevance of the *Bacillus subtilis* extracellular matrix in the avoidance of colonization by Pcl cells. The same experiment in King's B medium, however, indicated that diffusible molecules were more relevant than the extracellular matrix in modulating the interaction dynamics (Figure 1A; Figure S1). Time-lapse microscopy allowed us to better characterize the sequence of events that progressively shift the interaction from antagonism to co-existence during a period of 4−5 days. Unexpectedly, we could not restrict the antagonism of Pcl toward Bamy to a single compound. Our current results show that the antagonistic effect of Pcl in the short term is most likely multifactorial and controlled by the GacA-GacS two-component system. This environmental sensing system regulates the expression of genes required for the production of secondary metabolites (e.g., siderophores, hydrogen cyanide, pyoluteorin, phenazines) and extracellular enzymes that influence motility, iron acquisition, biofilm formation, and other aspects of primary metabolism in closely related *Pseudomonas* strains (Hassan et al., 2010; Heeb and Haas, 2001; Wang et al., 2013; Yan et al., 2018). In fact, this two-component system

Figure 5. Bamy *glpK* mutants suffer pleiotropic changes protecting from antibiosis of Pcl
(A) Time-course pairwise interactions between Pcl and GlpK^{S166L} (top) and ΔglpK (bottom) at 24, 48, and 72 h. Scale bars, 5 mm.
(B) Time-lapse microscopy analysis of the pairwise interaction between Pcl and GlpK^{S166L} during 48 h. Scale bars, 2 mm.
(C) CLSM time-course experiment of the interaction area between Pcl and GlpK^{S166L} using PI (red fluorescence) to identify cell death of GlpK^{S166L}. Images show the leading edge of the GlpK^{S166L} colony. Scale bars, 40 μm.
(D) Measurement of PI fluorescence emitted by GlpK^{S166L} during the interaction with Pcl as shown in (C). Error bars represent SD. n = 3.
(E) Differentially expressed genes between Bamy and GlpK^{S166L} at 24 h clustered into different metabolic pathways. Larger circles indicate the main KEGG pathways, which are surrounded by arrows pointing to smaller circles that represent the differentially expressed genes. The color of the arrows indicates induction (red) or repression (blue). The color of the circles differentiates pathways involved in metabolism (light blue) from those not involved in metabolism (light yellow).

CellPress
OPEN ACCESS

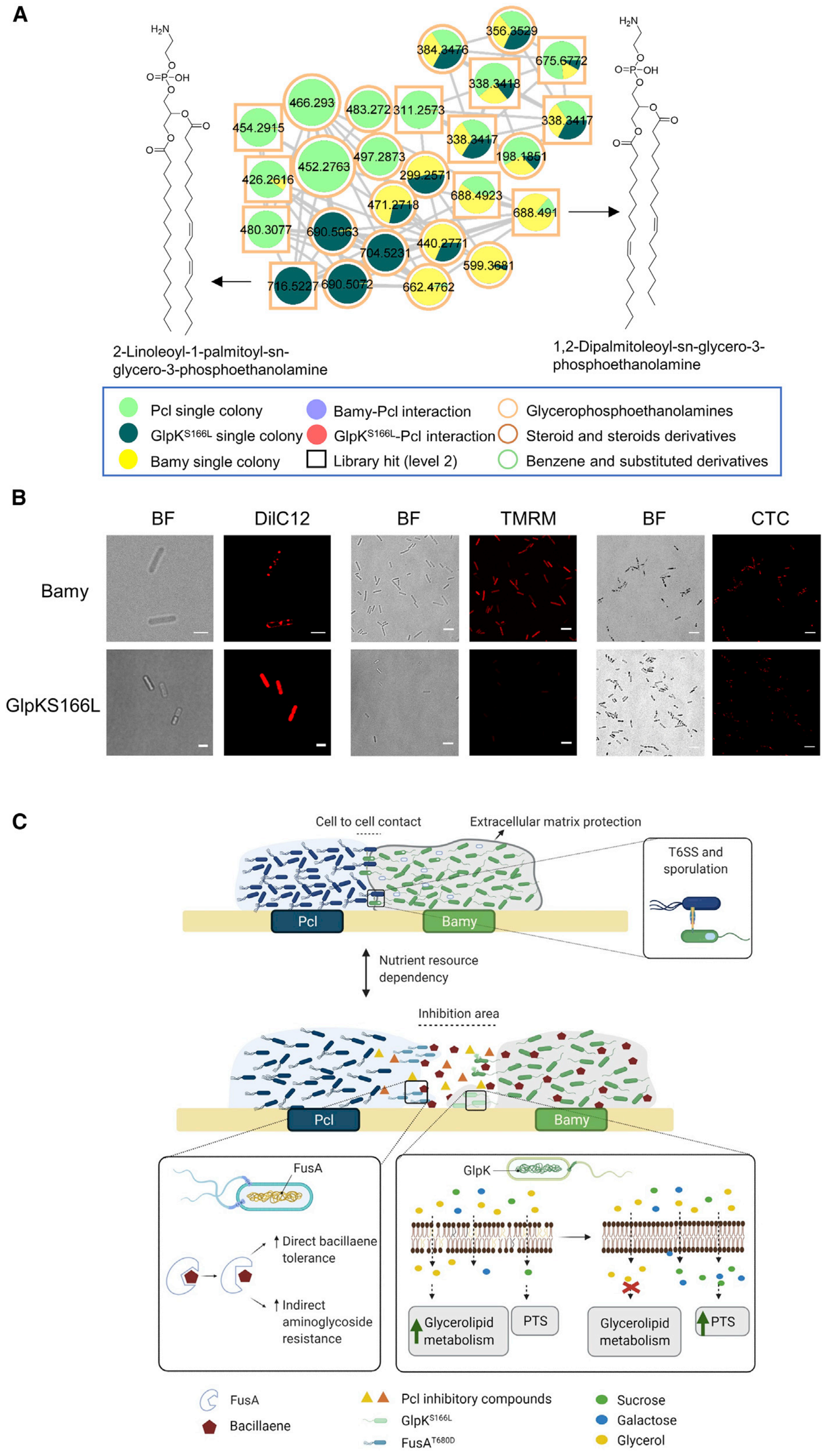

(legend on next page)

recurrently accumulates random mutations given its broad influence in the bacterial physiology and energy consumption (Bull et al., 2001; Duffy and Défago, 2000). Contrary to the combinatorial inhibition displayed by Pcl, we identified bacillaene as the most important molecule produced by Bamy that contributes to the initial antagonistic interaction. Bacillaene has been proposed to be a bacteriostatic molecule that protects *Bacillus* from predation by *Myxococcus xanthus* (Müller et al., 2015) and kills *Serratia plymuthica*, facilitating its engulfment by *B. subtilis* (Ogran et al., 2019). Although the arrest of protein synthesis has been suggested as its mode of action, this possibility has not been clarified (Patel et al., 1995). In this study, we have demonstrated that the bacteriostatic effect of bacillaene confers protection to the Bamy colony by impeding the expansion of the Pcl colony without massive cell death. Based on our mutational and *in silico* analyses, we concluded that bacillaene's mechanism of action (similar to that of fusidic acid; Besier et al., 2003) depends on an interaction with elongation factor G (FusA), which leads to translation inhibition. Consistently, a typical evolutionary resistance strategy mediated by modification of the antibiotic's target (Lambert, 2005), e.g., via point mutations in Pcl *fusA*, reduces the interaction with bacillaene, causing resistance to this molecule and indirectly to aminoglycosides that inhibit protein synthesis by targeting ribosomal subunits (Figure 6C).

The antagonism of Pcl based on multiple compounds might explain the fact that predominant spontaneous mutants of Bamy do not rely on a specific antibiotic target but in the gene *glpK* encoding the GlpK. The GlpK enzyme catalyzes the first step in the glycerolipid pathway converting glycerol into glycerol-3-phosphate, a modification that ensures the entrance of this carbon source in the catabolic pathways and promotes the formation of glycerolipids. Mutations of *glpK* have been also found in *Mycobacterium tuberculosis* in response to long antibiotic treatments; however, these are transient, and the mechanism behind the antibiotic tolerance remains unexplained (Bellerose et al., 2019; Safi et al., 2019). Others have speculated that GlpK may have an alternative role as a transcriptional regulator (Safi et al., 2019). The notorious transcriptional changes observed upon deletion of *glpK* affect complementary metabolic pathways oriented to the uptake of alternative carbon sources and obtaining energy but also metabolic pathways related with lipid metabolism, motility, sporulation, and signaling (Figure 5E; Table S5). These changes are evidence of the complementary role of GlpK. Based on this global deregulation, we hypothesize a diversified strategy led by GlpK to overcome antagonism in nutritional competitive scenarios (Figure 6C), supporting the recent ecological concept where new antibiotic resistance strategies point to the importance of mutations in metabolic genes (Lopatkin et al., 2021). First, GlpK causes the induction of sporulation and transcriptional changes in carbon source acquisition genes. This finding suggests its adaptation to utilizing alternative carbon sources, which transiently could help to overcome the presence of toxic molecules produced by competitors, and to the evolution of the interaction from the fight for nutrients and space to the bacterial co-existence (Ghoul and Mitri, 2016; Hibbing et al., 2010; Turcotte et al., 2012). Second, the changes in the lipid composition and the increase of rigidity of the cell membrane would lead to reduced permeability and thus entrance of antimicrobials (Bessa et al., 2018; Mingeot-Leclercq and Décout, 2016) (Figure 6; Table S4). Third, the induction of multidrug ATP-binding cassette transporters, type VII secretion system (T7SS), and penicillin-binding proteins could suggest their involvement in antimicrobial and toxic resistance as has been previously reported (Kengmo Tchoupa et al., 2020; Macheboeuf et al., 2006; Poole, 2007; Sun et al., 2014). Our findings, together with those obtained in human pathogens (Bellerose et al., 2019; Safi et al., 2019), highlight an important role of GlpK not only in antibiotic resistance but in bacterial adaptation to interactions in specific niches, from humans to plants.

Taken together, we propose a model where the initial antagonism for nutrients and space evolves toward a situation where WT and mutant genotypes seem to coexist in the same niche in a stable association of mixed subpopulations with mutual benefits for species survival (D'Souza and Kost, 2016). The participation of other bacterial structures (T6SS or extracellular matrix [ECM]) most likely are affected by the spatial effect imposed by the topology or chemical nature of the host, and would complementarily determine the final organization of the bacterial community. We conclude that the appearance of mutant subpopulations with different adaptability strategies is based on the metabolic specialization, and the involvement of chemical interactions finally determines the population size and the evolution toward mixed

Figure 6. Mutations in *glpK* provoke changes in membrane rigidity and permeability

(A) Glycerophosphoethanolamine cluster, obtained by mass spectrometry analysis using LC-MS/MS and feature-based molecular networking, showing modifications in the abundance of different glycerophosphoethanolamine compounds between Pcl, Bamy, and GlpK^{S166L}. Each metabolite is represented by a circle and they are connected according to the mass fragmentation patterns. Chemical structures of annotated features are based on spectral matches to GNPS libraries, as representative of these molecular families. Border color indicates ClassyFire classification. The sizes of the compounds are directly related to their abundance in the metabolome. Squares indicate a library hit level 2 through GNPS, while circles indicate unknown compounds based on GNPS.

(B) Membrane staining of Bamy and GlpK^{S166L} with (left panel) DilC12 dye to analyze differences in membrane fluidity (scale bars, 2 μm), (middle panel) TMRM straining for membrane potential analysis (scale bars, 5 μm), and (right panel) CTC staining to measure respiration changes (scale bars, 5 μm). For each experiment and sample, at least three fields of view were measured.

(C) Schematic representation of the proposed interaction between *Bacillus* and *Pseudomonas* depending on critical factors, e.g., nutrients and distance. (Upper part) When bacterial populations are in contact, T6SS, sporulation, and the extracellular matrix play important roles in the interaction. (Bottom part) When populations are physically separated, the production of secondary metabolites is critical for the evolution of the interaction. Mutations in *Pseudomonas fusA* permit adaptation to *Bacillus*-produced bacillaene, thereby increasing resistance to bacillaene and aminoglycoside antibiotics. Mutations in *Bacillus glpK* provoke a wide array of transcriptional and metabolic changes, an increase in bacterial membrane rigidity, and a reduction of membrane potential resulting in increased antibiotic tolerance.

and stable communities rather than the complete extinction of the competitor.

STAR★METHODS

Detailed methods are provided in the online version of this paper and include the following:

- KEY RESOURCES TABLE
- RESOURCE AVAILABILITY
  - Lead contact
  - Materials availability
  - Data and code availability
- EXPERIMENTAL MODEL AND SUBJECT DETAILS
- METHOD DETAILS
  - *Pseudomonas chlororaphis* mutants
  - *Bacillus amyloliquefaciens* mutants
  - Construction of fluorescence labeling strains
  - Pairwise interactions
  - Generation and screening of mini-Tn5 mutants of Pcl that do not inhibit Bamy
  - Whole-genome transcriptomic analysis
  - Isolation of spontaneous mutants and whole-genome sequencing
  - Compound purification
  - Minimum inhibitory concentration (MIC) assays
  - Matrix-assisted laser desorption ionization mass spectrometry imaging (MALDI-MSI)
  - Liquid chromatography-tandem mass spectrometry (LC-MS/MS)
  - Data analysis and MS/MS network analysis
  - Docking and *in silico* analysis of proteins
  - Membrane staining with DilC12
  - Membrane potential and respiration assays
- QUANTIFICATION AND STATISTICAL ANALYSIS
  - Statistical analysis

SUPPLEMENTAL INFORMATION

Supplemental information can be found online at https://doi.org/10.1016/j.celrep.2021.109449.

ACKNOWLEDGMENTS

We thank Saray Morales Rojas for technical support, the Ultrasequencing Unit of the SCBI-UMA for RNA sequencing, and the Bioinformatics Unit of GENYO (Granada, Spain) for the analytical treatment of the data. We are grateful to Prof. Francisco M. Cazorla for kindly providing the wild-type strain PCL1606, suggestions, and comments. This work was supported by grants from an ERC Starting Grant (BacBio 637971) and Plan Nacional de I+D+i of the Ministerio de Economía y Competitividad and the Ministerio de Ciencia e Innovación (AGL2016-78662-R and PID2019-107724GB-I00). C.M.-S. is funded by the program Juan de la Cierva Incorporación (IJC2018-036923-I). A.I.P.-L. is funded by the program FPU (FPU19/00289), JAE-Intro 2019 (JAEINT 19 00269), and the program Plan Propio de Investigación y Transferencia from Universidad de Málaga. D.P. was supported by the German Research Foundation (DFG) with Grant PE 2600/1. A.M.C.-R. and P.C.D. were supported by National Science Foundation Grant IOS-1656481 and National Institutes of Health (NIH) Grant 1DP2GM137413-01. P.C.D. was supported by the Gordon and Betty Moore Foundation through Grant GBMF7622 and the NIH (P41 GM103484, R03 CA211211, R01 GM107550).

AUTHOR CONTRIBUTIONS

D.R. conceived the study; D.R. and C.M.-S. designed the experiments; C.M.-S. performed the main experimental work; C.M.-S. and J.P. performed and designed the confocal microscopy work and data analysis; D.V.-C. performed the docking analyses; L.D.-M. and C.M.-S. performed the RNA sequencing and genomic data analyses; C.M.-S., S.S.-T., and A.I.P.-L. constructed strains; C.M.-S., D.P., P.C.D., and A.M.C.-R. performed MALDI-MSI and LC-MS/MS analyses; D.R. and C.M.-S. wrote the manuscript; and A.d.V., D.P., A.M.C.-R., and P.C.D. contributed critically to writing and editing the final version of the manuscript.

DECLARATION OF INTERESTS

The authors declare no competing interests.

Received: February 18, 2021
Revised: May 18, 2021
Accepted: July 2, 2021
Published: July 27, 2021

REFERENCES

Anders, S., and Huber, W. (2010). Differential expression analysis for sequence count data. Genome Biol. *11*, R106.

Andersson, D.I., and Hughes, D. (2014). Microbiological effects of sublethal levels of antibiotics. Nat. Rev. Microbiol. *12*, 465–478.

Arrebola, E., Tienda, S., Vida, C., de Vicente, A., and Cazorla, F.M. (2019). Fitness features involved in the biocontrol interaction of *Pseudomonas chlororaphis* with host plants: The case study of PcPCL1606. Front. Microbiol. *10*, 719.

Bastian, M., Heymann, S., and Jacomy, M. (2009). Gephi: an open source software for exploring and manipulating networks (International AAAI Conference on Weblogs and Social Media).

Bauer, M.A., Kainz, K., Carmona-Gutierrez, D., and Madeo, F. (2018). Microbial wars: Competition in ecological niches and within the microbiome. Microb. Cell *5*, 215–219.

Bellerose, M.M., Baek, S.-H., Huang, C.-C., Moss, C.E., Koh, E.-I., Proulx, M.K., Smith, C.M., Baker, R.E., Lee, J.S., Eum, S., et al. (2019). Common variants in the glycerol kinase gene reduce tuberculosis drug efficacy. MBio *10*, e00663-19.

Benoit, I., van den Esker, M.H., Patyshakuliyeva, A., Mattern, D.J., Blei, F., Zhou, M., Dijksterhuis, J., Brakhage, A.A., Kuipers, O.P., de Vries, R.P., and Kovács, Á.T. (2015). *Bacillus subtilis* attachment to *Aspergillus niger* hyphae results in mutually altered metabolism. Environ. Microbiol. *17*, 2099–2113.

Berlanga-Clavero, M.V., Molina-Santiago, C., de Vicente, A., and Romero, D. (2020). More than words: The chemistry behind the interactions in the plant holobiont. Environ. Microbiol. *22*, 4532–4544.

Bernal, P., Llamas, M.A., and Filloux, A. (2018). Type VI secretion systems in plant-associated bacteria. Environ. Microbiol. *20*, 1–15.

Besier, S., Ludwig, A., Brade, V., and Wichelhaus, T.A. (2003). Molecular analysis of fusidic acid resistance in *Staphylococcus aureus*. Mol. Microbiol. *47*, 463–469.

Bessa, L.J., Ferreira, M., and Gameiro, P. (2018). Evaluation of membrane fluidity of multidrug-resistant isolates of *Escherichia coli* and *Staphylococcus aureus* in presence and absence of antibiotics. J. Photochem. Photobiol. B *181*, 150–156.

Blin, K., Shaw, S., Steinke, K., Villebro, R., Ziemert, N., Lee, S.Y., Medema, M.H., and Weber, T. (2019). antiSMASH 5.0: Updates to the secondary metabolite genome mining pipeline. Nucleic Acids Res. *47* (*W1*), W81–W87.

Bolard, A., Plésiat, P., and Jeannot, K. (2018). Mutations in gene *fusA1* as a novel mechanism of aminoglycoside resistance in clinical strains of *Pseudomonas aeruginosa*. Antimicrob. Agents Chemother. *62*, e01835-17.

Bryan, L.E., and Kwan, S. (1983). Roles of ribosomal binding, membrane potential, and electron transport in bacterial uptake of streptomycin and gentamicin. Antimicrob. Agents Chemother. *23*, 835–845.

Bull, C.T., Duffy, B., Voisard, C., Défago, G., Keel, C., and Haas, D. (2001). Characterization of spontaneous gacS and gacA regulatory mutants of *Pseudomonas fluorescens* biocontrol strain CHAO. Antonie van Leeuwenhoek *79*, 327–336.

Calderón, C.E., Ramos, C., de Vicente, A., and Cazorla, F.M. (2015). Comparative genomic analysis of *Pseudomonas chlororaphis* PCL1606 reveals new insight into antifungal compounds involved in biocontrol. Mol. Plant Microbe Interact. *28*, 249–260.

Cámara-Almirón, J., Navarro, Y., Díaz-Martínez, L., Magno-Pérez-Bryan, M.C., Molina-Santiago, C., Pearson, J.R., de Vicente, A., Pérez-García, A., and Romero, D. (2020). Dual functionality of the amyloid protein TasA in *Bacillus* physiology and fitness on the phylloplane. Nat. Commun. *11*, 1859.

Cazorla, F.M., Duckett, S.B., Bergström, E.T., Noreen, S., Odijk, R., Lugtenberg, B.J., Thomas-Oates, J.E., and Bloemberg, G.V. (2006). Biocontrol of avocado dematophora root rot by antagonistic *Pseudomonas fluorescens* PCL1606 correlates with the production of 2-hexyl 5-propyl resorcinol. Mol. Plant Microbe Interact. *19*, 418–428.

Chen, X.H., Koumoutsi, A., Scholz, R., and Borriss, R. (2009a). More than anticipated - production of antibiotics and other secondary metabolites by *Bacillus amyloliquefaciens* FZB42. J. Mol. Microbiol. Biotechnol. *16*, 14–24.

Chen, X.H., Koumoutsi, A., Scholz, R., Schneider, K., Vater, J., Süssmuth, R., Piel, J., and Borriss, R. (2009b). Genome analysis of *Bacillus amyloliquefaciens* FZB42 reveals its potential for biocontrol of plant pathogens. J. Biotechnol. *140*, 27–37.

Chen, H.J., Hung, W.C., Tseng, S.P., Tsai, J.C., Hsueh, P.R., and Teng, L.J. (2010). Fusidic acid resistance determinants in *Staphylococcus aureus* clinical isolates. Antimicrob. Agents Chemother. *54*, 4985–4991.

Cornforth, D.M., and Foster, K.R. (2013). Competition sensing: The social side of bacterial stress responses. Nat. Rev. Microbiol. *11*, 285–293.

D'Souza, G., and Kost, C. (2016). Experimental evolution of metabolic dependency in bacteria. PLoS Genet. *12*, e1006364.

da Silva, R.R., Wang, M., Nothias, L.F., van der Hooft, J.J.J., Caraballo-Rodríguez, A.M., Fox, E., Balunas, M.J., Klassen, J.L., Lopes, N.P., and Dorrestein, P.C. (2018). Propagating annotations of molecular networks using in silico fragmentation. PLoS Comput. Biol. *14*, e1006089.

Deveau, A., Gross, H., Palin, B., Mehnaz, S., Schnepf, M., Leblond, P., Dorrestein, P.C., and Aigle, B. (2016). Role of secondary metabolites in the interaction between *Pseudomonas fluorescens* and soil microorganisms under iron-limited conditions. FEMS Microbiol. Ecol. *92*, fiw107.

Djoumbou Feunang, Y., Eisner, R., Knox, C., Chepelev, L., Hastings, J., Owen, G., Fahy, E., Steinbeck, C., Subramanian, S., Bolton, E., et al. (2016). ClassyFire: Automated chemical classification with a comprehensive, computable taxonomy. J. Cheminform. *8*, 61.

Duffy, B.K., and Défago, G. (2000). Controlling instability in gacS-gacA regulatory genes during inoculant production of *Pseudomonas fluorescens* biocontrol strains. Appl. Environ. Microbiol. *66*, 3142–3150.

Dührkop, K., Fleischauer, M., Ludwig, M., Aksenov, A.A., Melnik, A.V., Meusel, M., Dorrestein, P.C., Rousu, J., and Böcker, S. (2019). SIRIUS 4: A rapid tool for turning tandem mass spectra into metabolite structure information. Nat. Methods *16*, 299–302.

Dührkop, K., Nothias, L.F., Fleischauer, M., Reher, R., Ludwig, M., Hoffmann, M.A., Petras, D., Gerwick, W.H., Rousu, J., Dorrestein, P.C., et al. (2021). Systematic classification of unknown metabolites using high-resolution fragmentation mass spectra. Nat. Biotechnol. *39*, 462–471.

Edelstein, A.D., Tsuchida, M.A., Amodaj, N., Pinkard, H., Vale, R.D., and Stuurman, N. (2014). Advanced methods of microscope control using μManager software. J. Biol. Methods *1*, e10.

Ernst, M., Kang, K.B., Caraballo-Rodríguez, A.M., Nothias, L.F., Wandy, J., Chen, C., Wang, M., Rogers, S., Medema, M.H., Dorrestein, P.C., and van der Hooft, J.J.J. (2019). MolNetEnhancer: Enhanced molecular networks by integrating metabolome mining and annotation tools. Metabolites *9*, 144.

Falgueras, J., Lara, A.J., Fernández-Pozo, N., Cantón, F.R., Pérez-Trabado, G., and Claros, M.G. (2010). SeqTrim: A high-throughput pipeline for pre-processing any type of sequence read. BMC Bioinformatics *11*, 38.

Ghequire, M.G.K., and De Mot, R. (2015). The tailocin tale: Peeling off phage tails. Trends Microbiol. *23*, 587–590.

Ghoul, M., and Mitri, S. (2016). The ecology and evolution of microbial competition. Trends Microbiol. *24*, 833–845.

Gibson, D.G., Young, L., Chuang, R.Y., Venter, J.C., Hutchison, C.A., 3rd, and Smith, H.O. (2009). Enzymatic assembly of DNA molecules up to several hundred kilobases. Nat. Methods *6*, 343–345.

Granato, E.T., Meiller-Legrand, T.A., and Foster, K.R. (2019). The evolution and ecology of bacterial warfare. Curr. Biol. *29*, R521–R537.

Grosdidier, S., Pons, C., Solernou, A., and Fernández-Recio, J. (2007). Prediction and scoring of docking poses with pyDock. Proteins *69*, 852–858.

Hanahan, D. (1983). Studies on transformation of *Escherichia coli* with plasmids. J. Mol. Biol. *166*, 557–580.

Hassan, K.A., Johnson, A., Shaffer, B.T., Ren, Q., Kidarsa, T.A., Elbourne, L.D., Hartney, S., Duboy, R., Goebel, N.C., Zabriskie, T.M., et al. (2010). Inactivation of the GacA response regulator in Pseudomonas fluorescens Pf-5 has far-reaching transcriptomic consequences. Environ. Microbiol. *12*, 899–915.

Heeb, S., and Haas, D. (2001). Regulatory roles of the GacS/GacA two-component system in plant-associated and other gram-negative bacteria. Mol. Plant Microbe Interact. *14*, 1351–1363.

Hibbing, M.E., Fuqua, C., Parsek, M.R., and Peterson, S.B. (2010). Bacterial competition: Surviving and thriving in the microbial jungle. Nat. Rev. Microbiol. *8*, 15–25.

Kengmo Tchoupa, A., Watkins, K.E., Jones, R.A., Kuroki, A., Alam, M.T., Perrier, S., Chen, Y., and Unnikrishnan, M. (2020). The type VII secretion system protects *Staphylococcus aureus* against antimicrobial host fatty acids. Sci. Rep. *10*, 14838.

Kloepper, J.W., Ryu, C.M., and Zhang, S. (2004). Induced systemic resistance and promotion of plant growth by *Bacillus* spp. Phytopathology *94*, 1259–1266.

Kuzyakov, Y., and Xu, X. (2013). Competition between roots and microorganisms for nitrogen: Mechanisms and ecological relevance. New Phytol. *198*, 656–669.

Lambert, P.A. (2005). Bacterial resistance to antibiotics: Modified target sites. Adv. Drug Deliv. Rev. *57*, 1471–1485.

Langmead, B., and Salzberg, S.L. (2012). Fast gapped-read alignment with Bowtie 2. Nat. Methods *9*, 357–359.

LeRoux, M., Peterson, S.B., and Mougous, J.D. (2015). Bacterial danger sensing. J. Mol. Biol. *427*, 3744–3753.

Li, H., Handsaker, B., Wysoker, A., Fennell, T., Ruan, J., Homer, N., Marth, G., Abecasis, G., and Durbin, R.; 1000 Genome Project Data Processing Subgroup (2009). The Sequence Alignment/Map format and SAMtools. Bioinformatics *25*, 2078–2079.

Lin, L., Ringel, P.D., Vettiger, A., Durr, L., and Basler, M. (2019). DNA Uptake upon T6SS-dependent prey cell lysis induces SOS response and reduces fitness of Acinetobacter baylyi. Cell Rep. *29*, 1633–1644.e4.

Lopatkin, A.J., Bening, S.C., Manson, A.L., Stokes, J.M., Kohanski, M.A., Badran, A.H., Earl, A.M., Cheney, N.J., Yang, J.H., and Collins, J.J. (2021). Clinically relevant mutations in core metabolic genes confer antibiotic resistance. Science *371*, eaba0862. https://doi.org/10.1126/science.aba0862.

Lopez, J., Ly, P.M., and Feldman, M.F. (2020). The tip of the VgrG spike is essential to functional type VI secretion system assembly in *Acinetobacter baumannii*. MBio *11*, e02761-19.

Lozano, G.L., Bravo, J.I., Garavito Diago, M.F., Park, H.B., Hurley, A., Peterson, S.B., Stabb, E.V., Crawford, J.M., Broderick, N.A., and Handelsman, J. (2019). Introducing THOR, a model microbiome for genetic dissection of community behavior. MBio *10*, e02846-18.

Macheboeuf, P., Contreras-Martel, C., Job, V., Dideberg, O., and Dessen, A. (2006). Penicillin binding proteins: Key players in bacterial cell cycle and drug resistance processes. FEMS Microbiol. Rev. *30*, 673–691.

Martínez-García, E., and de Lorenzo, V. (2012). Transposon-based and plasmid-based genetic tools for editing genomes of gram-negative bacteria. Methods Mol. Biol. *813*, 267–283.

Matas, I.M., Lambertsen, L., Rodríguez-Moreno, L., and Ramos, C. (2012). Identification of novel virulence genes and metabolic pathways required for

full fitness of *Pseudomonas savastanoi* pv. savastanoi in olive (*Olea europaea*) knots. New Phytol. *196*, 1182–1196.

Mingeot-Leclercq, M.-P., and Décout, J.-L. (2016). Bacterial lipid membranes as promising targets to fight antimicrobial resistance, molecular foundations and illustration through the renewal of aminoglycoside antibiotics and emergence of amphiphilic aminoglycosides. MedChemComm *7*, 586–611.

Molina-Santiago, C., Daddaoua, A., Gómez-Lozano, M., Udaondo, Z., Molin, S., and Ramos, J.L. (2015). Differential transcriptional response to antibiotics by *Pseudomonas putida* DOT-T1E. Environ. Microbiol. *17*, 3251–3262.

Molina-Santiago, C., Cordero, B.F., Daddaoua, A., Udaondo, Z., Manzano, J., Valdivia, M., Segura, A., Ramos, J.L., and Duque, E. (2016). *Pseudomonas putida* as a platform for the synthesis of aromatic compounds. Microbiology (Reading) *162*, 1535–1543.

Molina-Santiago, C., Pearson, J.R., Navarro, Y., Berlanga-Clavero, M.V., Caraballo-Rodriguez, A.M., Petras, D., García-Martín, M.L., Lamon, G., Haberstein, B., Cazorla, F.M., et al. (2019). The extracellular matrix protects *Bacillus subtilis* colonies from *Pseudomonas* invasion and modulates plant co-colonization. Nat. Commun. *10*, 1919.

Müller, S., Strack, S.N., Ryan, S.E., Kearns, D.B., and Kirby, J.R. (2015). Predation by *Myxococcus xanthus* induces *Bacillus subtilis* to form spore-filled megastructures. Appl. Environ. Microbiol. *81*, 203–210.

Niehaus, L., Boland, I., Liu, M., Chen, K., Fu, D., Henckel, C., Chaung, K., Miranda, S.E., Dyckman, S., Crum, M., et al. (2019). Microbial coexistence through chemical-mediated interactions. Nat. Commun. *10*, 2052.

Nothias, L.F., Petras, D., Schmid, R., Dührkop, K., Rainer, J., Sarvepalli, A., Protsyuk, I., Ernst, M., Tsugawa, H., Fleischauer, M., et al. (2020). Feature-based molecular networking in the GNPS analysis environment. Nat. Methods *17*, 905–908.

Ogran, A., Yardeni, E.H., Keren-Paz, A., Bucher, T., Jain, R., Gilhar, O., and Kolodkin-Gal, I. (2019). The plant host induces antibiotic production to select the most-beneficial colonizers. Appl. Environ. Microbiol. *85*, e00512-19.

Parsek, M.R., and Greenberg, E.P. (2005). Sociomicrobiology: The connections between quorum sensing and biofilms. Trends Microbiol. *13*, 27–33.

Patel, P.S., Huang, S., Fisher, S., Pirnik, D., Aklonis, C., Dean, L., Meyers, E., Fernandes, P., and Mayerl, F. (1995). Bacillaene, a novel inhibitor of procaryotic protein synthesis produced by *Bacillus subtilis*: Production, taxonomy, isolation, physico-chemical characterization and biological activity. J. Antibiot. (Tokyo) *48*, 997–1003.

Perault, A.I., Chandler, C.E., Rasko, D.A., Ernst, R.K., Wolfgang, M.C., and Cotter, P.A. (2020). Host adaptation predisposes *Pseudomonas aeruginosa* to type VI secretion system-mediated predation by the *Burkholderia cepacia* complex. Cell Host Microbe *28*, 534–547.e3.

Pérez-Martínez, I., Zhao, Y., Murillo, J., Sundin, G.W., and Ramos, C. (2008). Global genomic analysis of *Pseudomonas savastanoi* pv. savastanoi plasmids. J. Bacteriol. *190*, 625–635.

Petras, D., Nothias, L.F., Quinn, R.A., Alexandrov, T., Bandeira, N., Bouslimani, A., Castro-Falcón, G., Chen, L., Dang, T., Floros, D.J., et al. (2016). Mass spectrometry-based visualization of molecules associated with human habitats. Anal. Chem. *88*, 10775–10784.

Pluskal, T., Castillo, S., Villar-Briones, A., and Oresic, M. (2010). MZmine 2: Modular framework for processing, visualizing, and analyzing mass spectrometry-based molecular profile data. BMC Bioinformatics *11*, 395.

Poole, K. (2007). Efflux pumps as antimicrobial resistance mechanisms. Ann. Med. *39*, 162–176.

Preibisch, S., Saalfeld, S., and Tomancak, P. (2009). Globally optimal stitching of tiled 3D microscopic image acquisitions. Bioinformatics *25*, 1463–1465.

Puri, A.W., Mevers, E., Ramadhar, T.R., Petras, D., Liu, D., Piel, J., Dorrestein, P.C., Greenberg, E.P., Lidstrom, M.E., and Clardy, J. (2018). Tundrenone: An atypical secondary metabolite from bacteria with highly restricted primary metabolism. J. Am. Chem. Soc. *140*, 2002–2006.

Rieusset, L., Rey, M., Muller, D., Vacheron, J., Gerin, F., Dubost, A., Comte, G., and Prigent-Combaret, C. (2020). Secondary metabolites from plant-associated *Pseudomonas* are overproduced in biofilm. Microb. Biotechnol. *13*, 1562–1580.

Robinson, M.D., McCarthy, D.J., and Smyth, G.K. (2010). edgeR: A Bioconductor package for differential expression analysis of digital gene expression data. Bioinformatics *26*, 139–140.

Rueden, C.T., Schindelin, J., Hiner, M.C., DeZonia, B.E., Walter, A.E., Arena, E.T., and Eliceiri, K.W. (2017). ImageJ2: ImageJ for the next generation of scientific image data. BMC Bioinformatics *18*, 529.

Safi, H., Gopal, P., Lingaraju, S., Ma, S., Levine, C., Dartois, V., Yee, M., Li, L., Blanc, L., Ho Liang, H.P., et al. (2019). Phase variation in *Mycobacterium tuberculosis glpK* produces transiently heritable drug tolerance. Proc. Natl. Acad. Sci. USA *116*, 19665–19674.

Schindelin, J., Arganda-Carreras, I., Frise, E., Kaynig, V., Longair, M., Pietzsch, T., Preibisch, S., Rueden, C., Saalfeld, S., Schmid, B., et al. (2012). Fiji: An open-source platform for biological-image analysis. Nat. Methods *9*, 676–682.

Smith, J.M., and Price, G.R. (1973). The logic of animal conflict. Nature *246*, 15–18.

Stempler, O., Baidya, A.K., Bhattacharya, S., Malli Mohan, G.B., Tzipilevich, E., Sinai, L., Mamou, G., and Ben-Yehuda, S. (2017). Interspecies nutrient extraction and toxin delivery between bacteria. Nat. Commun. *8*, 315.

Sun, S., Selmer, M., and Andersson, D.I. (2014). Resistance to β-lactam antibiotics conferred by point mutations in penicillin-binding proteins PBP3, PBP4 and PBP6 in *Salmonella enterica*. PLoS ONE *9*, e97202.

Taber, H.W., Mueller, J.P., Miller, P.F., and Arrow, A.S. (1987). Bacterial uptake of aminoglycoside antibiotics. Microbiol. Rev. *51*, 439–457.

Turcotte, M.M., Corrin, M.S.C., and Johnson, M.T.J. (2012). Adaptive evolution in ecological communities. PLoS Biol. *10*, e1001332.

Turner, K.H., Everett, J., Trivedi, U., Rumbaugh, K.P., and Whiteley, M. (2014). Requirements for *Pseudomonas aeruginosa* acute burn and chronic surgical wound infection. PLoS Genet. *10*, e1004518.

Tyc, O., de Jager, V.C.L., van den Berg, M., Gerards, S., Janssens, T.K.S., Zaagman, N., Kai, M., Svatos, A., Zweers, H., Hordijk, C., et al. (2017). Exploring bacterial interspecific interactions for discovery of novel antimicrobial compounds. Microb. Biotechnol. *10*, 910–925.

Valli, R.X.E., Lyng, M., and Kirkpatrick, C.L. (2020). There is no hiding if you Seq: Recent breakthroughs in *Pseudomonas aeruginosa* research revealed by genomic and transcriptomic next-generation sequencing. J. Med. Microbiol. *69*, 162–175.

Vieira, S., Sikorski, J., Dietz, S., Herz, K., Schrumpf, M., Bruelheide, H., Scheel, D., Friedrich, M.W., and Overmann, J. (2020). Drivers of the composition of active rhizosphere bacterial communities in temperate grasslands. ISME J. *14*, 463–475.

Wang, D., Lee, S.H., Seeve, C., Yu, J.M., Pierson, L.S., 3rd, and Pierson, E.A. (2013). Roles of the Gac-Rsm pathway in the regulation of phenazine biosynthesis in *Pseudomonas chlororaphis* 30-84. MicrobiologyOpen *2*, 505–524.

Wang, M., Carver, J.J., Phelan, V.V., Sanchez, L.M., Garg, N., Peng, Y., Nguyen, D.D., Watrous, J., Kapono, C.A., Luzzatto-Knaan, T., et al. (2016). Sharing and community curation of mass spectrometry data with Global Natural Products Social Molecular Networking. Nat. Biotechnol. *34*, 828–837.

Wei, Z., Gu, Y., Friman, V.P., Kowalchuk, G.A., Xu, Y., Shen, Q., and Jousset, A. (2019). Initial soil microbiome composition and functioning predetermine future plant health. Sci. Adv. *5*, eaaw0759.

Yan, Q., Lopes, L.D., Shaffer, B.T., Kidarsa, T.A., Vining, O., Philmus, B., Song, C., Stockwell, V.O., Raaijmakers, J.M., McPhail, K.L., et al. (2018). Secondary metabolism and interspecific competition affect accumulation of spontaneous mutants in the GacS-GacA regulatory system in *Pseudomonas protegens*. MBio *9*, e01845-17.

Yang, J., and Zhang, Y. (2015). I-TASSER server: New development for protein structure and function predictions. Nucleic Acids Res. *43* (*W1*), W174–W181.

STAR★METHODS

KEY RESOURCES TABLE

REAGENT or RESOURCE	SOURCE	IDENTIFIER
Bacterial and virus strains		
Table S6		N/A
Chemicals, peptides, and recombinant proteins		
Fusidic acid	Sigma-Aldrich	Cat#F0756-1G
Kanamycin	Sigma-Aldrich	Cat#B5264-250MG
Gentamicin	Sigma-Aldrich	Cat#G1914-250MG
Ampicillin	Sigma-Aldrich	Cat#A9393
Pyochelin	USBiological life sciences	Cat#L19111952
Spectinomycin	Sigma-Aldrich	Cat#S0692
Critical commercial assays		
Gibson Assembly Master Mix	NewEngland Biolabs	Cat#E2611L
DiIC12	ThermoFisher	Cat#D383
TMRM reagent	Invitrogen	Cat#T668
Propidium Iodide	ThermoFisher	Cat#P1304MP
Deposited data		
LC-MS/MS data	https://massive.ucsd.edu/ProteoSAFe/static/massive.jsp	Massive MSV000085326
	Wang et al., 2016	Feature-based molecular networking analysis
	Nothias et al., 2020	https://gnps.ucsd.edu/ProteoSAFe/status.jsp?task=574c0acbea58405e80e1c0dc526bc903
	Ernst et al., 2019	MolNetEnhancer
		https://gnps.ucsd.edu/ProteoSAFe/status.jsp?task=15e9aa6189e04c859b685eb0eb0f6089
RNaseq data		GEO database GEO: GSE161161
Genome sequences		GenBank PRJNA680227
Oligonucleotides		
Table S7		N/A
Software and algorithms		
ImageJ	National Institute of health	https://imagej.nih.gov/ij/
i-TASSER	Zhang Lab	https://zhanglab.ccmb.med.umich.edu/I-TASSER/
FlexImaging 3.0	Bruker	https://fleximaging.com/
Mzmine 2.30	Pluskal et al., 2010	http://mzmine.github.io/
GNPS	Wang et al., 2016	https://gnps.ucsd.edu/ProteoSAFe/static/gnps-splash.jsp
SeqtrimNext v2.1.3	Falgueras et al., 2010	https://github.com/dariogf/SeqtrimNext
Samtools	Li et al., 2009	http://www.htslib.org/
Bowtie2	Langmead and Salzberg, 2012	http://bowtie-bio.sourceforge.net/bowtie2/index.shtml
Gephi	Bastian et al., 2009	https://gephi.org

RESOURCE AVAILABILITY

Lead contact

Further information and requests for resources and reagents should be directed to and will be fulfilled by the lead contact, Diego Romero (diego_romero@uma.es).

Materials availability

Plasmids generated in this study are available in BacBio Lab. You can contact Diego Romero (diego_romero@uma.es).

This study did not generate new unique reagents.

Data and code availability

Data availability

RNaseq data have been deposited at GEO database and are publicly available as of the date of publication. Accession numbers are listed in the Key Resources Table.

Genome sequences data have been deposited at NCBI and are publicly available as of the date of publication. Accession numbers are listed in the Key Resources Table.

LC-MS/MS data have been deposited at Mass Spectrometry Interactive Virtual Environment (MassIVE) at https://massive.ucsd.edu/ProteoSAFe/static/massive.jsp and are publicly available as of the date of publication. Accession numbers are listed in the Key Resources Table.

Code availability

This paper does not report original code.

Post-publication availability of data and code

Any additional information required to reanalyze the data reported in this paper is available from the lead contact upon request.

EXPERIMENTAL MODEL AND SUBJECT DETAILS

A complete list of the bacterial strains used in this study is shown in Table S6. Routinely, bacterial cells were grown in liquid lysogeny broth (LB) medium at 30°C (Pcl and Bamy) or 37°C (*E. coli*) with shaking on an orbital platform. When necessary, antibiotics were added to the media at appropriate concentrations. Strains and plasmids were constructed using standard methods.

METHOD DETAILS

Pseudomonas chlororaphis mutants

Chromosomal deletions of Pcl mutants were performed using the I-SceI method (Martínez-García and de Lorenzo, 2012; Molina-Santiago et al., 2016) in which upstream and downstream segments of homologous DNA are separately amplified and then joined to a previously digested pEMG vector using Gibson Assembly Master Mix (Gibson et al., 2009). The oligonucleotide sequences are shown in Table S7. The resulting plasmid was then electroporated into Pcl1606. After selection for positive clones, the pSEVA628S I-SceI expression plasmid was also electroporated, and kanamycin (Km)-sensitive clones were analyzed via PCR to verify the deletions. The pSEVA628S plasmid was cured via growth without selective pressure, and its loss confirmed by sensitivity to 60 μg ml^{-1} gentamicin and colony PCR screening as described by Martinez-Garcia and de Lorenzo (Martínez-García and de Lorenzo, 2012).

Bacillus amyloliquefaciens mutants

Bamy strains with *glpK* mutations were obtained as follows. Approximately 1500-base pair genomic regions upstream and downstream of the genes of interest were amplified from Bamy FZB42 chromosomal DNA. The two gel-purified double-stranded DNA fragments were linked by a Km resistance cassette and then ligated into pMAD. The linearized plasmids were integrated into the genome of Bamy via double-crossover recombination, yielding the Bamy knockout mutants.

Construction of fluorescence labeling strains

The fluorescence labeling plasmid pKM008V was constructed for Bamy strains. Briefly, the P_{veg} promoter fragment (300 bp) was extracted from pBS1C3 via EcoRI and HindIII digestion, purified, and cloned into the plasmid pKM008, which was previously digested with the same restriction enzymes. We used P_{veg} as it is considered a constitutive promoter in Bamy. pKM008V was then linearized and transformed into Bamy via natural competence, and transformants were selected by plating on LB plates supplemented with spectinomycin (100 μg ml^{-1}).

Pairwise interactions

Bamy and Pcl strains were routinely spotted 0.7 cm apart on King's B agar plates using 2 μl of cell suspension at an OD_{600} of 0.5. M9 agar medium supplemented with glycerol as sole carbon source was used as indicated in the text. Plates were incubated at 30°C, and were images taken at different time points. For confocal microscopy time-course experiments, 0.7 μl of cell suspension was spotted at distance of 0.5 cm onto 1.3-mm thick LB agar supplemented with propidium iodide in 35-mm glass-bottomed dishes suitable for confocal microscopy (Ibidi). Temperature was maintained at 28 °C during the time-course using the integrated microscope incubator. Acquisitions were performed using an inverted Leica SP5 confocal microscope with a 25 × NA 0.95 NA IR APO long working distance water immersion objective. Image processing and three-dimensional (3D) visualization were performed using ImageJ/FIJI (Rueden et al., 2017; Schindelin et al., 2012) and Imaris version 7.6 (Bitplane).

Colony time-lapse videos were acquired using a Nikon Ti inverted microscope equipped with DIC brightfield illumination and a 4x Plan NA 0.1 dry objective. A stage-top incubation system with an incorporated digital temperature sensor (Okolab) was used to maintain the temperature at 28°C. Time-lapse images were acquired using open-source Micromanager Software version 2.0 beta (Edelstein et al., 2014) and a Hamamatsu Orca R2 monochrome camera set to 2 × 2 binning and 4 ms exposure. Mosaic acquisition was performed with a 10% field overlap using the multi-dimensional acquisition module and two-step autofocus (JAF H&P; 1st step size 2.0, 1st step number 1.0; 2nd step size 0.2, 2nd step number 5; threshold 0.02 and crop ratio 0.2). Time-lapse images were typically captured over 2–4 days at 20-minute intervals. Mosaic merging was performed using FIJI and the Grid/Collection stitching plugin (Preibisch et al., 2009). Flatfield correction was performed using the Stack Normalizer plugin (Joachim Walter; https://imagej.nih.gov/ij/plugins/normalizer.html) using a Gaussian-filtered time zero image as reference.

Generation and screening of mini-Tn5 mutants of Pcl that do not inhibit Bamy

Pcl mini-Tn5 transposon mutants were constructed as described by Matas et al. (Matas et al., 2012) with minor modifications. The pool of pUTminiTn5Km1 vectors was transferred from *E. coli* S17 λpir to Pcl via plate conjugation mating as previously described (Pérez-Martínez et al., 2008). The constructed random transposition collection consisted of 28 96-well microtiter trays with a total of 2688 Pcl mutants. For screening, a lawn of Bamy was inoculated onto King's B solid medium and dried for 15 minutes at room temperature. Next, 1 μl of each Pcl mutants was individually spotted on the plates followed by incubation at 30°C for 24 hours. Pcl mutants that did not cause an inhibition halo around them were selected. Genomic DNA was extracted from the Pcl mutant strains using the Jet Flex Extraction Kit (Genomed, Löhne, Germany) according to the manufacturer's instructions. To determine transposon insertion sites, genomic DNA was digested with PstI or XbaI and ligated into pBluescript II SK digested with the same restriction enzyme. Ligation reactions were used to transform DH5α by heat shock (Hanahan, 1983), and single Km-resistant colonies were selected. Plasmids were purified and DNA regions flanking transposons were sequenced by Macrogen (Madrid, Spain) using primer P7 and the sequences were analyzed using BLASTn.

Whole-genome transcriptomic analysis

Single colonies of Pcl, Bamy, and the GlpK^{S166L} mutant were grown overnight on solid LB medium at 30°C and spotted on King's B medium as single colonies or as interactions as previously described for 24 hours. Next, cells were collected and stored at −80°C. All assays were performed in duplicate. For disruption of single colonies and interactions, collected cells were resuspended in BirnBoim A75 and lysozyme was added followed by incubation at 37°C for 15 minutes. Next, the suspensions were centrifuged, the pellets were resuspended in Trizol, and total RNA was extracted as indicated by the manufacturer. DNA removal was carried out via treatment with Nucleo-Spin RNA Plant (Macherey-Nagel). Integrity and quality of total RNA was assessed with an Agilent 2100 Bioanalyzer (Agilent Technologies). Removal of rRNA was performed using RiboZero rRNA removal (bacteria) kit from Illumina, and 100-bp single-end reads libraries were prepared using a TruSeq Stranded Total RNA Kit (Illumina). Libraries were sequenced using a NextSeq550 sequencer (Illumina).

The raw reads were pre-processed with SeqTrimNext (Falgueras et al., 2010) using the specific NGS technology configuration parameters. This pre-processing removes low-quality, ambiguous and low-complexity stretches, linkers, adapters, vector fragments, and contaminated sequences while keeping the longest informative parts of the reads. SeqTrimNext also discarded sequences below 25 bp. Subsequently, clean reads were aligned and annotated using the Pcl and Bamy reference genomes with Bowtie2 (Langmead and Salzberg, 2012) in BAM files, which were then sorted and indexed using SAMtools v1.484(Li et al., 2009). Uniquely localized reads were used to calculate the read number value for each gene via Sam2counts (https://github.com/vsbuffalo/sam2counts). Differentially expressed genes (DEGs) were analyzed via DEgenes Hunter, which provides a combined p value calculated (based on Fisher's method) using the nominal p values provided by from edgeR (Robinson et al., 2010) and DEseq2 (Anders and Huber, 2010). This combined p value was adjusted using the Benjamini-Hochberg (BH) procedure (false discovery rate approach) and used to rank all the obtained DEGs. For each gene, combined p value < 0.05 and log2-fold change > 1 or < -1 were considered as the significance threshold (for Bamy versus GlpK^{S166L} the log2-fold change was fixed in > 2 or < -2). The annotated DEGs were used to identify the Gene Ontology functional categories and KEGG pathways. Gephi software (https://gephi.org) was used to generate the DEG networks.

Isolation of spontaneous mutants and whole-genome sequencing

Bamy and Pcl strains were spotted 0.7 cm apart onto King's B agar plates using 2 μl of cell suspension at an OD_{600} of 0.5. Plates were incubated at 30°C for 5–6 days. Next, small Bamy colonies and Pcl overgrowing subzones that were observed in the inhibition area were isolated and tested in co-culture assays (described below). Seven Bamy isolates and three Pcl isolates were used for whole-genome sequencing. Sequencing libraries were prepared using the PCRfree TrueSeq Kit from Illumina. 250-bp paired-end reads were sequenced using an Illumina MiSeq platform. Illumina raw reads, in fastq format, were trimmed using SeqtrimNext v2.1.3 to remove low-quality and low-complexity sequences, adapters, polyA tails, and several contaminant sequences. Useful reads were then mapped against their respective reference genomes (Bamy GenBank: NC_009725; Pcl GenBank: CP011110) to prepare data for subsequent variant calling using BWA configured with options –B 20 –A 30 –O 30 –E 3 to ensure high-quality alignments and accurate mapping scoring to prevent false variant calling. In the next step, a pileup file from each mapped sample was created using Samtools mpileup with options –BQ 26 –q 30 –d 10000000, as recommened by the Samtools official documentation

for obtaining accurate results. Finally, mutations and InDels were independently called with VarScan2 using the pileup of bam files. Genome sequences were deposited at NCBI under Bioproject PRJNA680227.

Compound purification

Pcl was grown on King's B plates for 24 hours. Next, both bacteria and solid media were obtained, and a methanol (MeOH) (LC-MS grade, Fisher) extraction was performed with 15 minutes of sonication before centrifugation. The extracted solution was filtered and diluted to 10% MeOH with water (LC-MS grade, Fisher). Extracts were then pre-fractionated in solid phase C18 resin. After stepwise elution with 20, 40, 60, 80, and 100% MeOH (LC-MS grade Fisher), fractions were assayed to identify inhibitory compounds. Fractions with higher inhibitory activity were subjected to semi-preparative HPLC purification using a 10 × 150 mm C18 column (XBridge Waters). For the mobile phase, we used a flow rate of 5 mL/min (solvent A: H2O + 0.1% formic acid (FA), solvent B: acetonitrile (ACN) + 0.1% FA). During the chromatographic separation, we applied a linear gradient from 0−1 min, 5% B, 1−10 min 5−50% B, 10−15 min 50−99% B, followed by a 5-minute washout phase at 99% B and a 5 minute re-equilibration phase at 5% B. 1-mL fractions were collected in deep well plates, and fractions of interest were then dried in a vacuum centrifuge (Centrivap, Labconco). Weight was recorded for all isolated compounds.

Minimum inhibitory concentration (MIC) assays

MIC assays were performed in liquid LB medium using the two-fold serial dilution test according to the guidelines of the Clinical and Laboratory Standards Institute (2003). The highest concentrations of the compounds were: fusidic acid (125000 μg ml^{-1}), pyochelin (500 μg ml^{-1}), HPR (1000 μg ml^{-1}), kanamycin (1000 μg ml^{-1}), gentamicin (1500 μg ml^{-1}), and ampicillin (10000 μg ml^{-1}). Experiments were carried out in triplicate and the MIC was determined as the lowest antibiotic concentration that inhibited growth by > 90%.

Matrix-assisted laser desorption ionization mass spectrometry imaging (MALDI-MSI)

To perform MALDI-MSI, a small section of King's B agar containing the cultured microorganisms (both in single colonies and in interactions) were cut and transferred to a MALDI MSP 96 anchor plate. Deposition of matrix (1:1 mixture of 2,5-dihydroxybenzoic acid and α-cyano-4-hydroxycinnamic acid) over the agar was performed using a 53-μm molecular sieve. Next, plates were dried at 37 °C for 4 hours. Images were collected before and after matrix deposition. Samples were analyzed using a Bruker Microflex MALDI-TOF mass spectrometer (Bruker Daltonics, Billerica, MA, USA) in positive reflectron mode, with 300 μm–400 μm laser intervals in X and Y directions, and a mass range of 100–3200 Da. Data were analyzed using FlexImaging 3.0 software (Bruker Daltonics, Billerica, MA, USA). The acquired spectra were normalized by dividing all the spectra by the mean of all data points (TIC normalization method). The resulting mass spectrum was then filtered manually in 0.25% (3.0 Da) increments assigning colors to the selected ions associated with the metabolites of interest.

Liquid chromatography-tandem mass spectrometry (LC-MS/MS)

Non-targeted LC-MS/MS analysis was performed on a Q-Exactive Quadrupole-Orbitrap mass spectrometer coupled to Vanquish ultra-high performance liquid chromatography (UHPLC) system (Thermo Fisher Scientific, Bremen, Germany) according to (Petras et al., 2016). 5 μL of the samples were injected for UHPLC separation on a C18 core-shell column (Kinetex, 50 × 2 mm, 1.8-um particle size, 100 A-pore size, Phenomenex, Torrance, CA, USA). For the mobile phase, we used a flow rate of 0.5 mL/min (solvent A: H_2O + 0.1% formic acid (FA), solvent B: acetonitrile (ACN) + 0.1% FA). During the chromatographic separation, we applied a linear gradient from 0–0.5 min, 5% B, 0.5–4 min 5%–50% B, 4–5 min 50%–99% B, followed by a 2-minute washout phase at 99% B and a 2-minute re-equilibration phase at 5% B. For positive mode MS/MS acquisition, the electrospray ionization (ESI) was set to a 35 L/min sheath gas flow, 10 L/min auxiliary gas flow, 2 L/min sweep gas flow, with a 400°C auxiliary gas temperature. The spray voltage was set to 3.5 kV with an inlet capillary of 250°C. The S-lens voltage was set to 50 V. MS/MS product ion spectra were acquired in data-dependent acquisition (DDA) mode. MS1 survey scans (150–1500 m/z) and up to 5 MS/MS scans per DDA duty cycle were measured with a resolution (R) of 17,500. The C-trap fill time was set to a maximum of 100 ms or until the AGC target of 5E5 ions was reached. The quadrupole precursor selection width was set to 1 m/z. Normalized collision energy was applied stepwise at 20, 30, and 40% with z = 1 as the default charge state. MS/MS scans were triggered with apex mode within 2–15 s from their first occurrence in a survey scan. Dynamic precursor exclusion was set to 5 s. Precursor ions with unassigned charge states and isotope peaks were excluded from MS/MS acquisition.

Data analysis and MS/MS network analysis

After LC-MS/MS acquisition, raw spectra were converted to .mzXML files using MSconvert (ProteoWizard). MS1 and MS/MS feature extraction was performed with Mzmine2.30(Pluskal et al., 2010). For MS1 spectra, an intensity threshold of 1E5 was used, and for MS/MS spectra, an intensity threshold of 1E3 was used. For MS1 chromatogram building, a 10-ppm mass accuracy and a minimum peak intensity of 5E5 was set. Extracted ion chromatograms (XICs) were deconvolved using the baseline cut-off algorithm at an intensity of 1E5. After chromatographic deconvolution, XICs were matched to MS/MS spectra within 0.02 m/z and 0.2-minute retention time windows. Isotope peaks were grouped and features from different samples were aligned with 10 ppm mass tolerance and 0.1-minute retention time tolerance. MS1 features without MS2 features assigned were filtered out the resulting matrix as well as

features that did not contain isotope peaks and that did not occur in at least three samples. After filtering, gaps in the feature matrix were filled with relaxed retention time tolerance at 0.2 minute but also 10 ppm mass tolerance. Finally, the feature table was exported as a .csv file, and corresponding MS/MS spectra exported as .mgf files. Contaminate features observed in blank samples were filtered, and only those with a relative abundance ratio blank to average lower than 50% were considered for further analysis.

For feature-based molecular networking and spectrum library matching, the .mgf file was uploaded to GNPS (https://gnps.ucsd.edu/ProteoSAFe/static/gnps-splash.jsp) (Wang et al., 2016). For molecular networking, the minimum cosine score was set to 0.7. The precursor ion mass tolerance was set to 0.01 Da, and the fragment ion mass tolerance was set to 0.01 Da. Minimum matched fragment peaks were set to 4, minimum cluster size was set to 1 (MS Cluster off), and library search minimum matched fragment peaks were set to 4. When analog searches were performed, the cosine score threshold was 0.7 and the maximum analog search mass difference was 100 *m/z*. Molecular networks were visualized with Cytoscape version 3.484.

To enhance the chemical structural information in the molecular network, information from *in silico* structure annotations from GNPS Library Search, Network Annotation Propagation were incorporated into the network using the GNPS MolNetEnhancer workflow (https://ccms-ucsd.github.io/GNPSDocumentation/molnetenhancer/) on the the GNPS website (https://gnps.ucsd.edu/ProteoSAFe/static/gnps-splash.jsp). Chemical class annotations were performed using the ClassyFire chemical ontology (da Silva et al., 2018; Djoumbou Feunang et al., 2016; Ernst et al., 2019; Wang et al., 2016).

Docking and *in silico* analysis of proteins

I-Tasser workspace (https://zhanglab.ccmb.med.umich.edu/I-TASSER/) was used for automated protein tertiary structure homology modeling of FusA (Yang and Zhang, 2015). To identify potential binding sites of bacillaene (PubChem ID: 25144999) and fusidic acid (PubChem ID: 3000226) to the Pcl *fusA*-encoded protein, automated molecular docking and thermodynamic analysis were performed using the web-based SwissDock program (www.swissdock.ch/docking) (Grosdidier et al., 2007). SwissDock predicts possible molecular interactions between a target protein and a small molecule based on the EADock DSS docking algorithm (Grosdidier et al., 2007). Docking was performed using the "Accurate" parameter with otherwise default parameters, with no region of interest defined (blind docking). Binding energies were estimated via CHARMM (Chemistry at HARvard Macromolecular Mechanics), a molecular simulation program implemented within SwissDock software. The most favorable energies are evaluated via FACTS (Fast Analytical Continuum Treatment of Solvation). Finally, the resulting energies were scored and ranked based on full fitness (kcal mol^{-1}), and the spontaneous binding was reported as the estimated Gibbs free energy ΔG (kcal mol^{-1}). Negative ΔG values support the assertion that the binding process is highly spontaneous. Modeling and docking results were visualized using UCSF Chimera v1.8 software.

Membrane staining with DiIC12

To detect regions of increased membrane fluidity, staining with DiIC12 (Thermo Fisher) staining was performed. Bamy strains were grown overnight on King's B plates and then resuspended in 1 mL dH_2O followed by addition of 1 $\mu g\ ml^{-1}$ DiIC12. 0.5% Benzylalcohol was added to positive control samples. Cells were incubated at 28°C for 3 hours and then washed four times and resuspended in dH_2O. Images were obtained by visualizing samples using a Leica SP5 confocal microscope with a 63x NA 1.3 Plan APO oil immersion objective and acquisitions with excitation at 651 nm. Image processing was performed using FIJI/ImageJ software. For each experiment, laser settings, scan speed, PMT detector gain, and pinhole aperture were kept constant for all acquired image stacks.

Membrane potential and respiration assays

Membrane potential was evaluated using the image-iT TMRM reagent (Invitrogen) following the manufacturer's instructions. Colonies grown at 28 °C on King's B solid medium were isolated at 24 hours and resuspended in 1X phosphate-buffered saline. As a control, samples were treated with 20 μM carbonyl cyanide m-chlorophenyl hydrazine (CCCP), a known protonophore and uncoupler of bacterial oxidative phosphorylation, prior to staining. TMRM was added to the bacterial suspensions to a final concentration of 100 nM, and the mixtures were incubated at 37 °C for 30 minutes. After incubation, the cells were immediately visualized by confocal laser scanning microscopy (CLSM) with excitation at 561 nm and emission detection between 576 and 683 nm.

Respiratory activity was measured using 5 mM CTC staining following the manufacturer's instructions. Colonies were grown as described above, and samples were incubated with CTC at 37°C for 30 minutes. After incubation, the cells were immediately visualized by CLSM with excitation at 561 nm and emission detection between 576 and 683 nm.

QUANTIFICATION AND STATISTICAL ANALYSIS

Statistical analysis

All results are presented as the mean ± SD. Microsoft Excel and GraphPad 9 were used for statistical analysis. Data were analyzed using one-way ANOVA and t test as indicated in the figure legends.

Cell Reports, Volume *36*

Supplemental information

Chemical interplay and complementary adaptative strategies toggle bacterial antagonism and co-existence

Carlos Molina-Santiago, David Vela-Corcía, Daniel Petras, Luis Díaz-Martínez, Alicia Isabel Pérez-Lorente, Sara Sopeña-Torres, John Pearson, Andrés Mauricio Caraballo-Rodríguez, Pieter C. Dorrestein, Antonio de Vicente, and Diego Romero

Supplementary data

Supplementary Figures

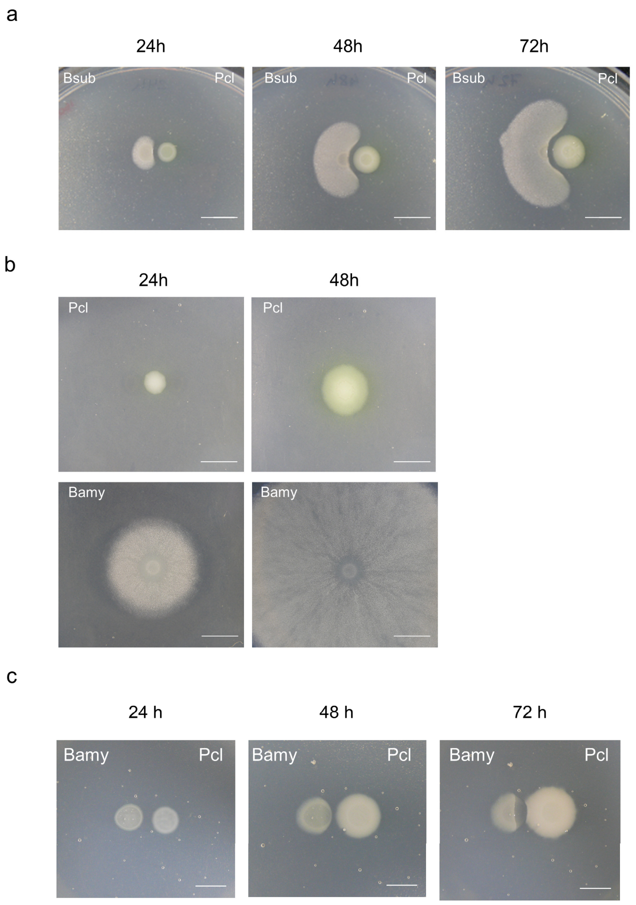

Figure S1. Pcl inhibits *B. subtilis* 3610 on King's B medium and Bamy on M9+glycerol. Related to Figure 1. a) Pairwise interaction time-lapse between *B. subtilis* 3610 (left) and Pcl (right) in King's B medium at 24, 48, and 72 h. Scale = 10 mm. b) Single colony growth of Pcl (top) and Bamy (bottom) on King's B medium at 24 and 48 h. c) Pairwise interaction time-lapse between Bamy (left) and Pcl (right) in M9 medium supplemented with glycerol as sole carbon source at 24, 48, and 72 h. Scale = 10 mm.

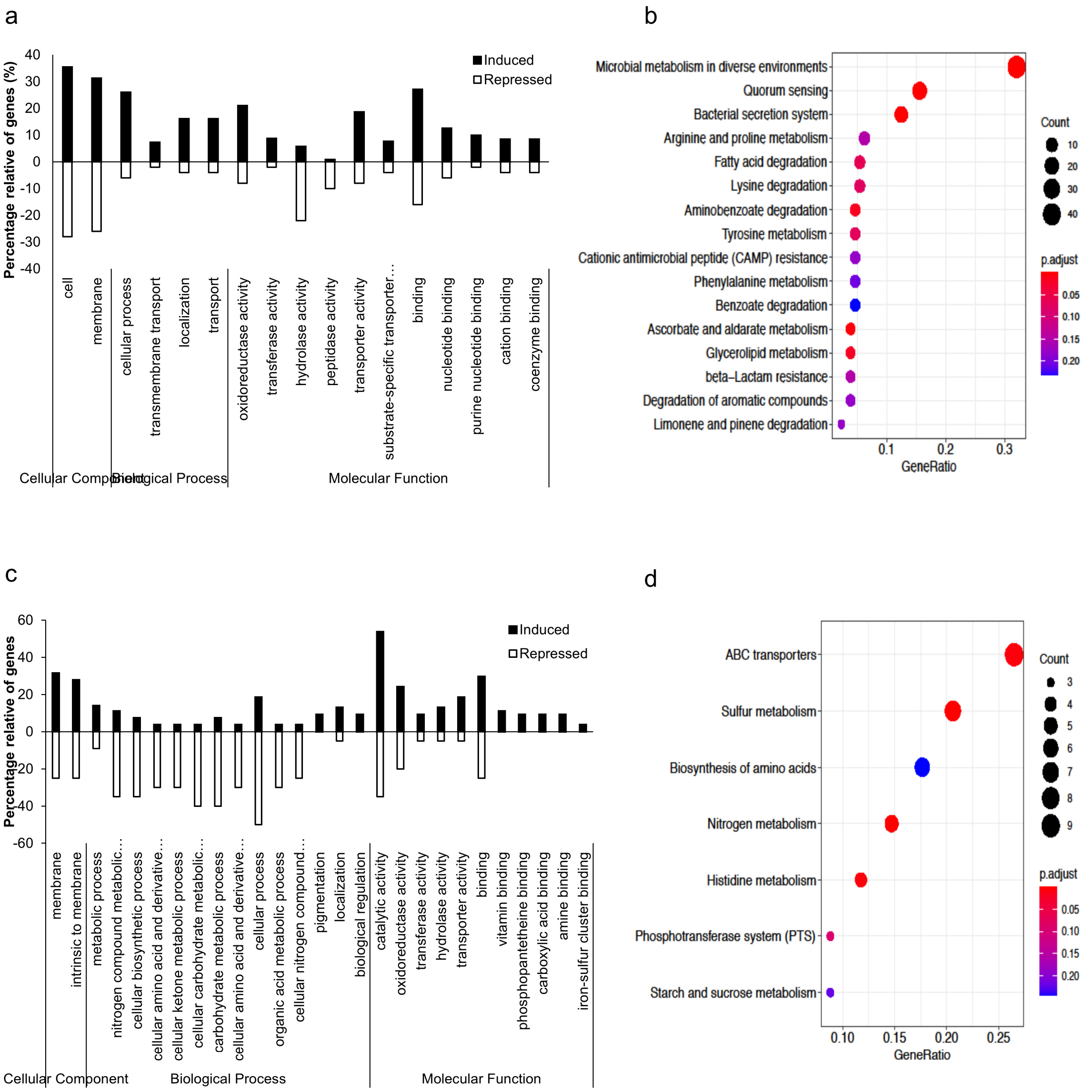

Figure S2. Transcriptomic changes during the interaction of Pcl with Bamy at 24 h. Related to Figure 2. a) GO terms of differentially expressed genes in Pcl. Black bars indicate induced GO terms and empty bars indicate repressed GO terms. b) KEGG pathways differentially expressed during the interaction in Pcl compared with control samples. c) KEGG pathways differentially expressed during the interaction in Bamy compared with control samples. d) GO terms of differentially expressed genes in Bamy. Black bars indicate induced GO terms and empty bars indicate repressed GO terms.

a

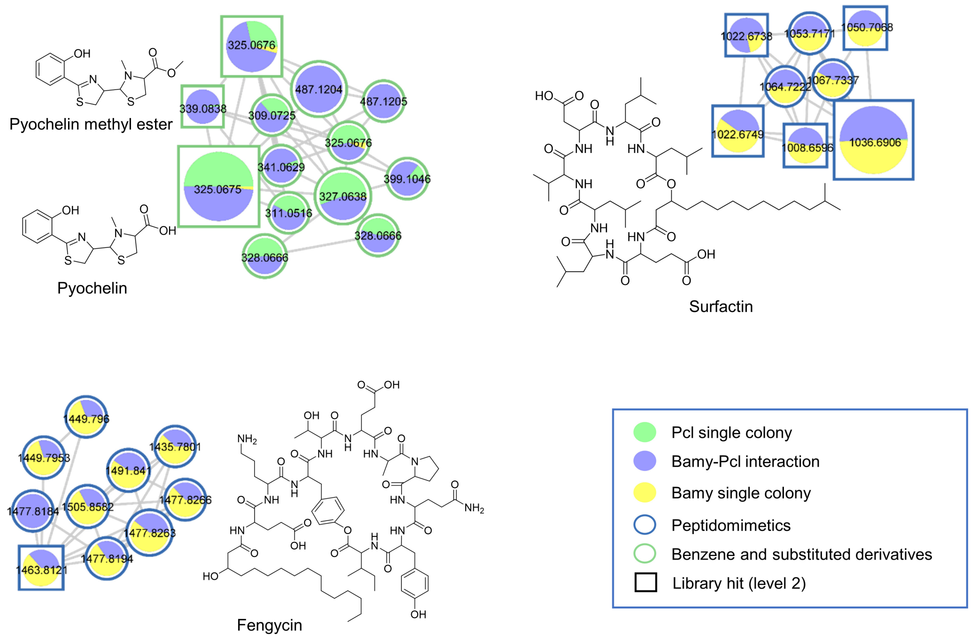

b

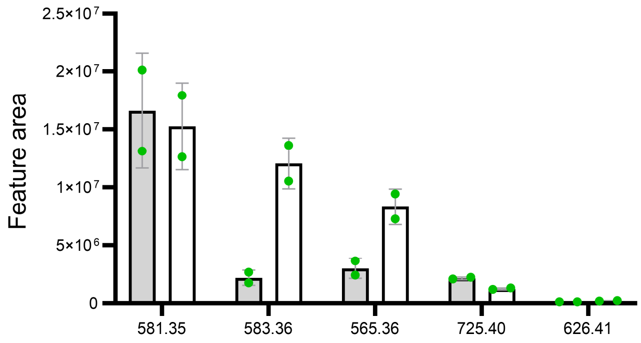

Figure S3. a) Molecular families of pyochelin, surfactin and fengycin detected from Bamy and Pcl growing alone and in interaction. Related to Figure 2. Results shown were obtained by mass spectrometry using LC-MS/MS and feature based molecular networking. Each metabolite is represented by a circle and they are connected according to the mass fragmentation patterns. The chemical structures of the annotated features are based on spectral matches in GNPS libraries representative of specific molecular families. Border

colors indicate ClassyFire classification. The sizes of the compounds are directly related to their abundance in the metabolome. Squares indicate a library hit level 2 through GNPS, and circles indicate unknown compounds based on GNPS searches. b) Bar plots show the quantification of the relative abundance of the selected molecules in Bamy growing alone and in interaction with Pcl. Grey bars represent the feature area of metabolites in Bamy single colonies, while empty bars represent the feature area of metabolites produced by Bamy during the interaction with Pcl. Statistical analysis done using t-test. Error bars indicate SD, *n* = 2.

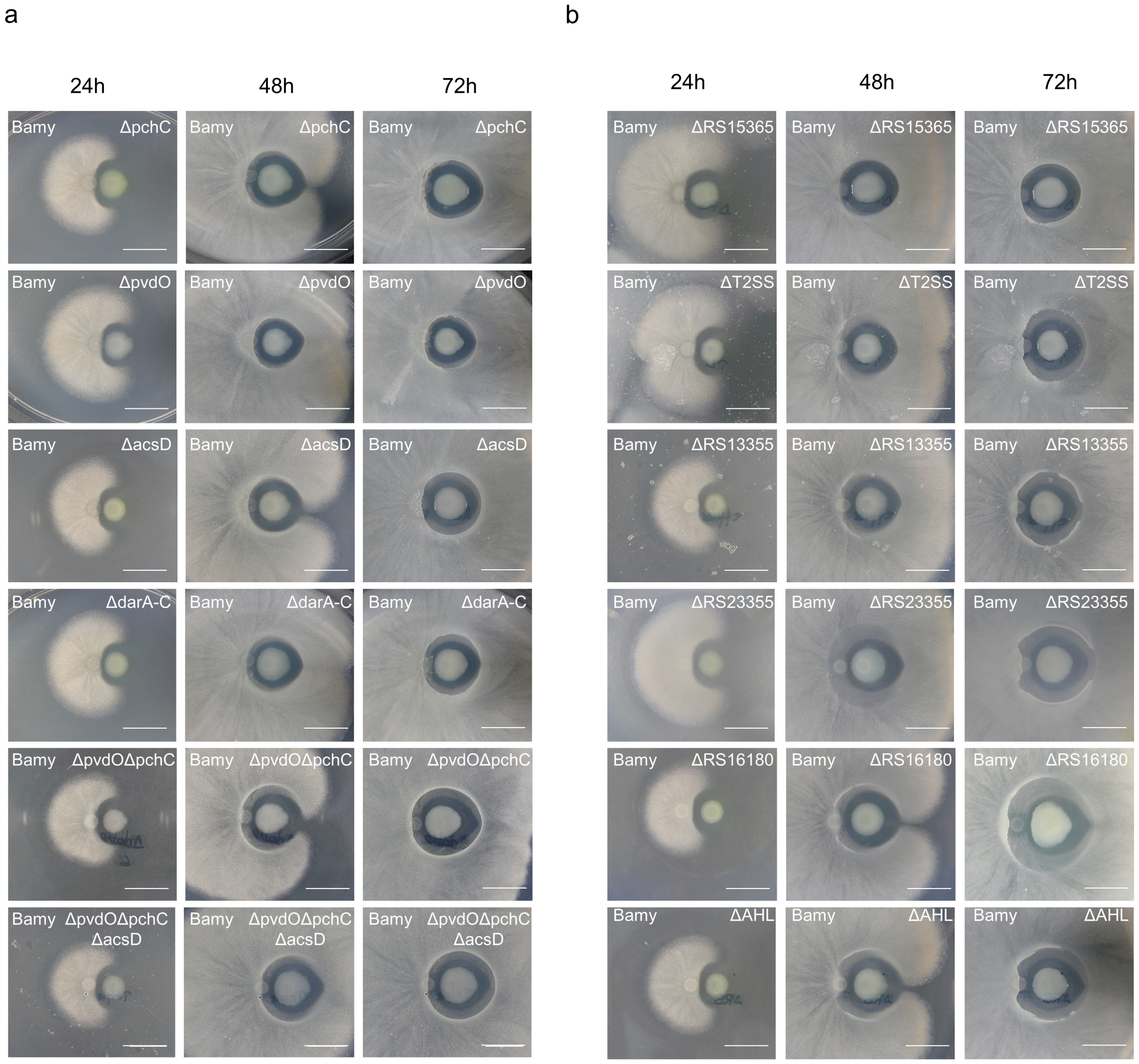

Figure S4. a) Time-course pairwise interactions between Bamy and Pcl mutants involving secondary metabolites on King's B medium at 24, 48, and 72 h. Related to Figure 2. Scale = 10 mm. ΔpvdO = pyoverdine mutant; ΔpchC = pyochelin mutant; ΔacsD = achromobactin mutant; ΔdarA-C = HPR mutant; ΔpvdOΔpchC = double mutant in pyoverdine and pyochelin; ΔpvdOΔpchCΔacsD = triple mutant in pyoverdine, pyochelin, and achromobactin. b) Time-course pairwise interactions between Bamy and Pcl mutants involving secondary metabolites, T2SS, and efflux pumps on King's B medium at 24, 48, and 72 h. Scale = 10 mm. ΔAHL = acyl-homoserine lactone mutant; ΔT2SS = type II secretion system mutant.

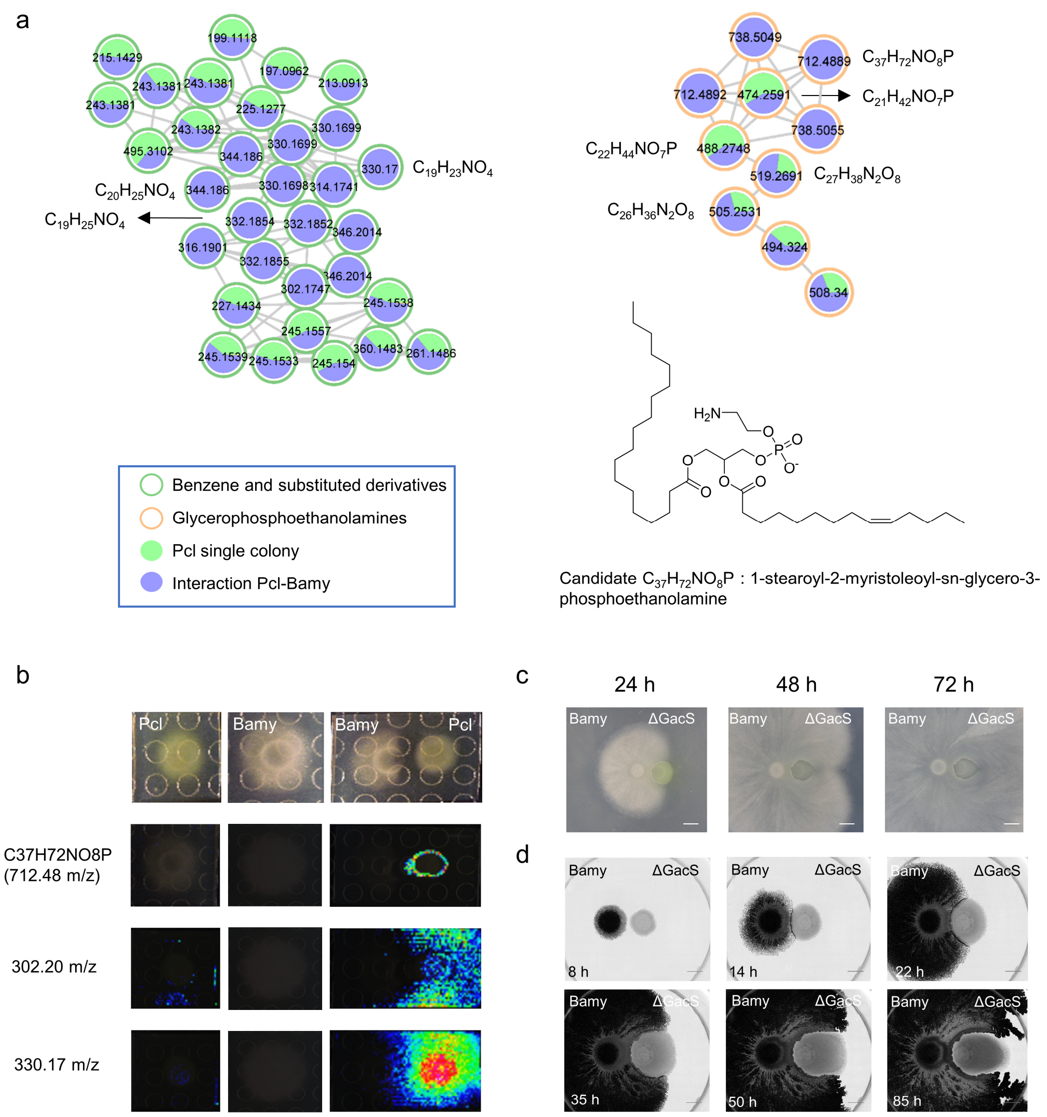

Figure S5. Candidate Pcl metabolites responsible for Bamy inhibition. Related to Figure 2. a) Molecular families of Pcl secondary metabolites mostly detected during the interaction with Bamy. Chemical structure of annotated features based on SIRIUS analyses, as representative of these molecular families. Border color indicates ClassyFire classification. The sizes of the compounds are directly related to their abundance in the metabolome. Squares indicate a library hit level 2 in GNPS, while circles indicate unknown compounds based on GNPS. b) MALDI-TOF-MSI heatmaps showing the spatial distribution of

representative metabolites indicated in panel a with m/z 712.48, 302.20 and 330.17. c) Pairwise interaction time lapse between Bamy (left) and *ΔGacS* (right) on King's B media at 24, 48, and 72 h. Scale = 5 mm. d) Time-lapse microscopy of the pairwise interaction between Bamy (left) and *ΔGacS* (right) during 85 h. Scale= 2 mm.

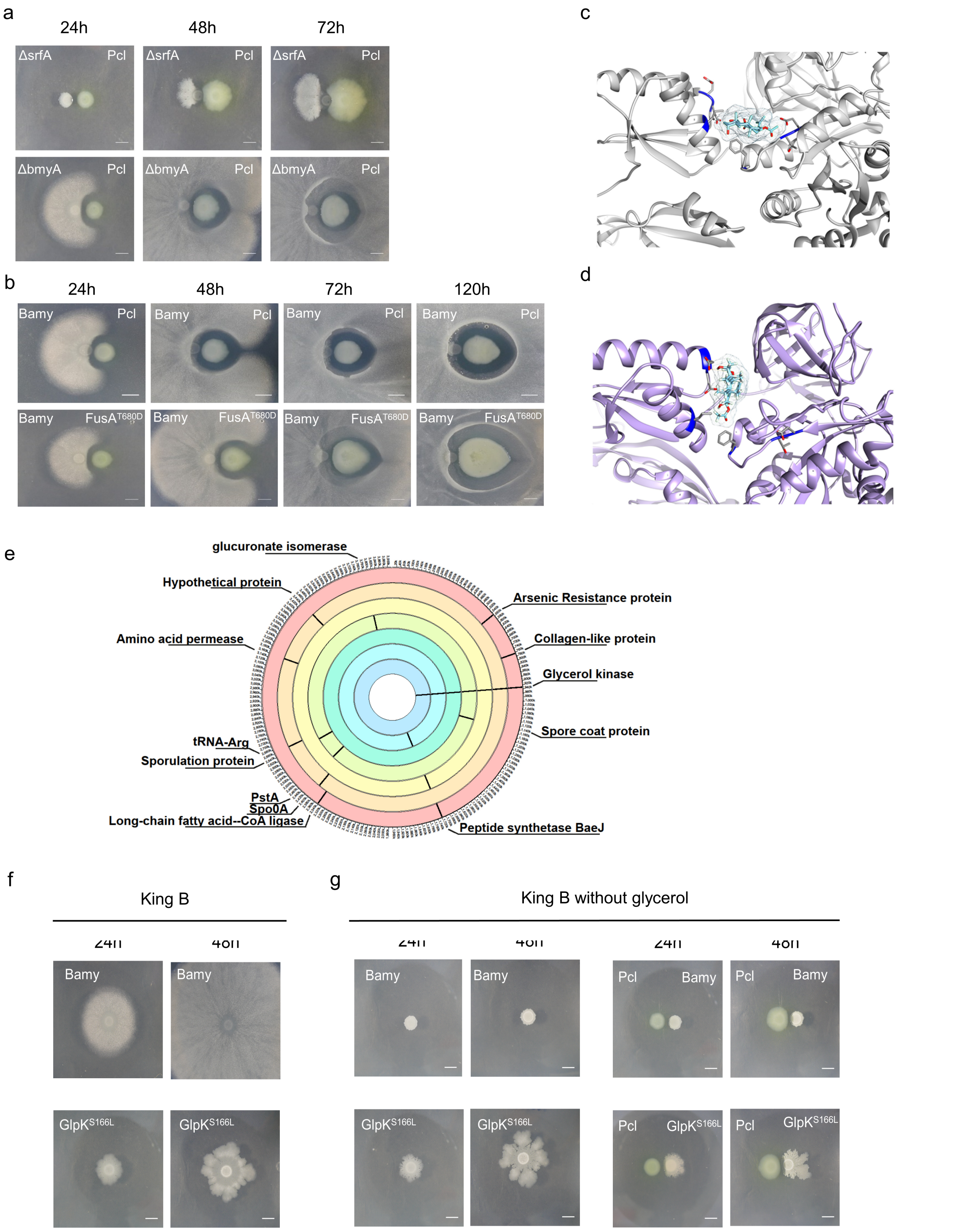

Figure S6. Related to Figures 3 and 4. a) Pairwise interactions between Pcl and Bamy mutants related to the secondary metabolites surfactin (*ΔsrfA*) (top) and bacillomycin (*ΔbmyA*) (bottom). Scale = 5 mm. b) Pairwise interactions between Bamy and Pcl WT (top)

or FusA^{T680D} (bottom) on King's B medium at 24, 48, 72 and 120 h. Scale = 5 mm. c and d) Molecular docking between fusidic acid and (c) FusA or (d) FusA^{T680D} showing differences in the binding pocket. e) Circular representation of the point mutations found in the genomes of Bamy clones with *glpK* mutations. Each ring represents one sequenced Bamy clone. f) and g) Growth differences in Bamy strains on King's B medium with and without glycerol. f) Single Bamy and GlpK^{S166L} colonies growing on King's B medium at 24 and 48 h. g) Single Bamy and GlpK^{S166L} colonies and their interactions with Pcl growing on King's B medium without glycerol at 24 and 48 h.

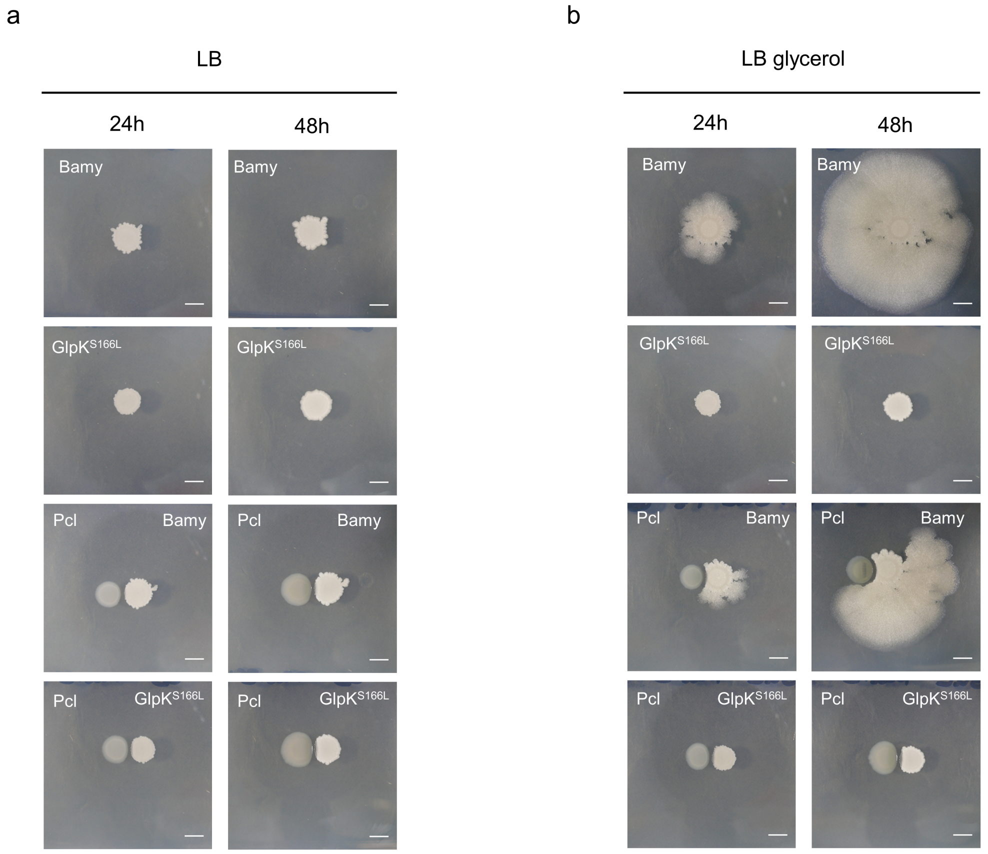

Figure S7. Growth differences in Bamy strains on LB medium with and without glycerol. Related to Figure 5. a) Single Bamy and GlpK^{S166L} colonies and their interactions with Pcl growing on LB B medium at 24 and 48 h. b) Single Bamy and GlpK^{S166L} colonies and their interactions with Pcl growing on LB medium supplemented with glycerol at 24 and 48 h. Scale = 5 mm.

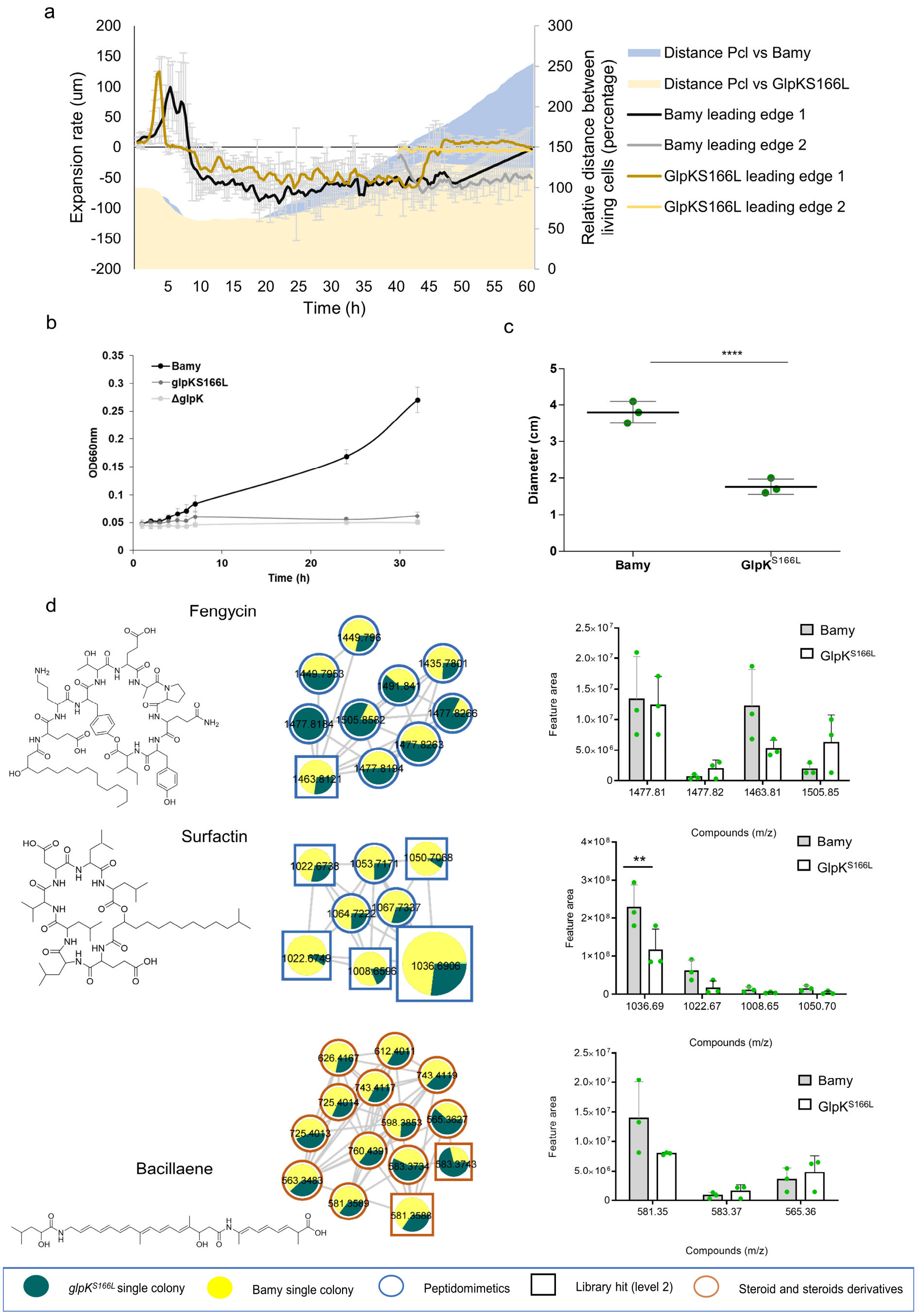

Figure S8. Phenotypic changes in a GlpK^{S166L} mutant. Related to Figures 5 and 6. a) Expansion rates and distance of the GlpK^{S166L} and Bamy leading edges during the interaction with Pcl. Brown and yellow lines represent GlpK^{S166L} leading edges 1 and 2. Black

and grey lines represent Bamy leading edges 1 and 2. Blue area represents the distance between the Bamy and Pcl populations during the entire interaction while the light orange area represents the distance between the GlpK^{S166L} and Pcl populations. Error bars represent SD. b) Growth curves of Bamy (black dots), GlpK^{S166L} (dark grey dots) and ΔglpK (grey dots) in M9 supplemented with 5 mM glycerol. c) Swarming motility reduction in a GlpK^{S166L} mutant. The plot represents the diameter of the colony. Statistical analysis done using t-test. ****P-value < 0.0001. Error bars represent SD, n = 3. d) Metabolomic changes found in GlpK^{S166L} in comparison with Bamy. Molecular families of the secondary metabolites fengycin, surfactin, and bacillaene. The chemical structures of annotated features are based on spectral matches to GNPS libraries as representative of these molecular families. Border color indicates ClassyFire classification. Yellow color represents relative abundance in Bamy, while dark green color represents relative abundance in GlpK^{S166L}. Bar plots show the quantification of the relative abundance of the selected molecules in the interactions in Bamy and GlpK^{S166L} strains. Grey bars represent Bamy feature area of metabolites, while empty bars represent GlpK^{S166L} feature area of metabolites. Statistical analysis done using t-test. **P-value < 0.01. Error bars represent SD, n = 23.

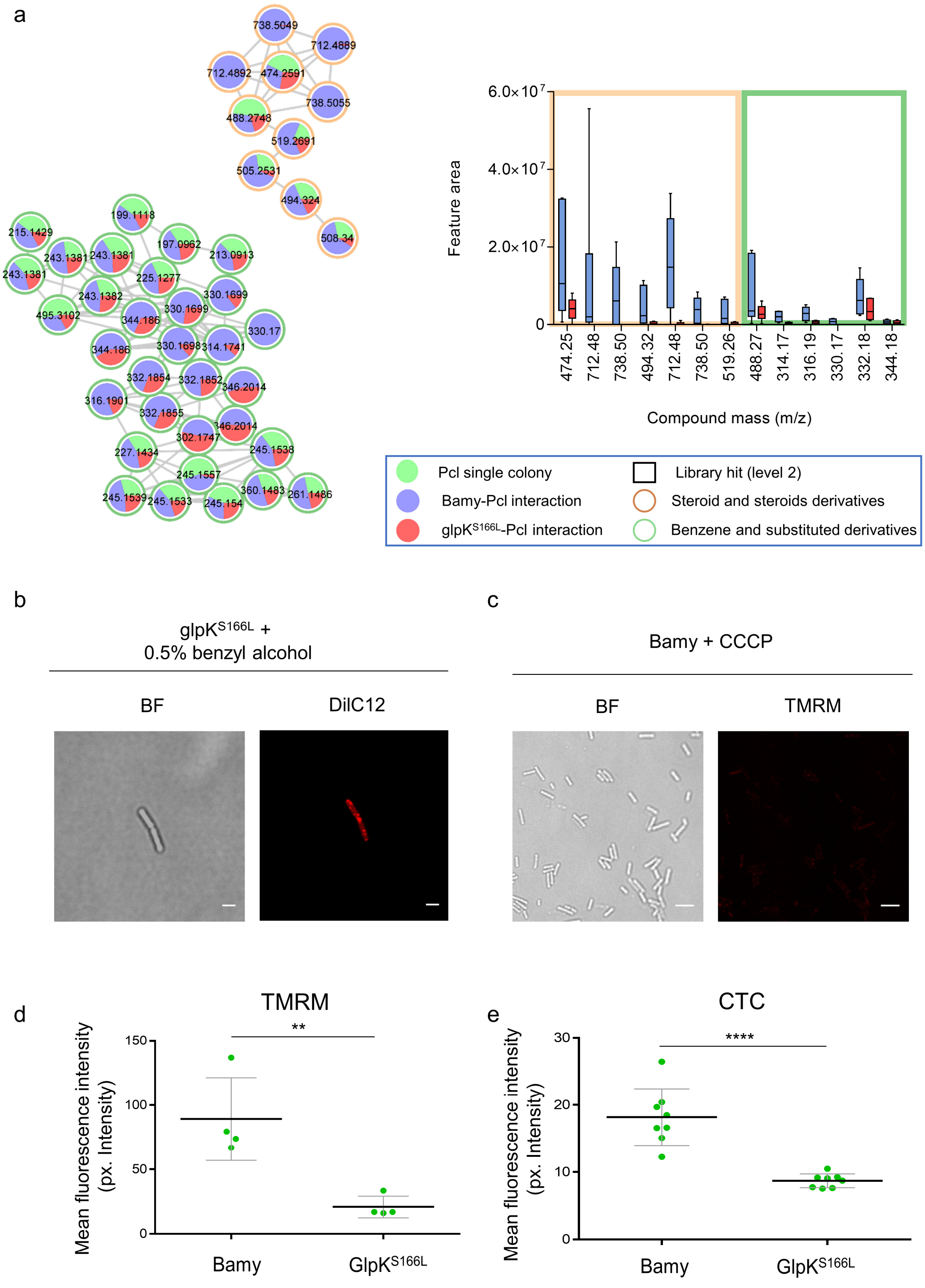

Figure S9. Metabolomic changes found in GlpK^{S166L} during the interaction with Pcl in comparison with the interaction of Bamy with Pcl. Related to Figure 6. a) Changes in the abundance of candidate molecular families of secondary metabolites produced by Pcl during

the interaction with Bamy and GlpK^{S166L}. The chemical structures of annotated features are based on spectral matches to GNPS libraries, as representative of these molecular families. Border color indicates ClassyFire classification. Right panel represents the quantification of the relative abundance of the selected molecules during Pcl-Bamy and Pcl-GlpK^{S166L} interactions. *n* = 6. Statistical test 2-way ANOVA. b) Membrane staining of GlpK^{S166L} with (left panel) DilC12 dye using 0.5% benzyl alcohol as positive control (scale = 2 μm). c) TMRM positive control experiment of Bamy supplemented with 20 μM CCCP (scale 5 μm). d) Quantification of the TMRM signal in Bamy and GlpK^{S166L}. *n* = 4. e) Quantification of the CTC signal in Bamy and GlpK^{S166L} cells. Error bars represent the SEM, *n* = 8. Statistical significance in the TMRM and CTC experiments was assessed via two-tailed independent t-tests at each time-point (***P-value* < 0.01).

Supplementary Tables

Table S3. Metabolites purified from a Pcl culture with MIC activity against Bamy. Related to Figure 2.

Mass (m/z)	Compound	MIC Bamy (µg/ml)
237.18	2-Hexyl-5-propyl-rescorcinol (HPR)	40
239.2	unknown	62.5
339.08	Pyochelin methyl ester	250
444.22	unknown	250

Table S4. MIC of PcI and *fusA* mutants to aminoglycoside antibiotics kanamycin (Km) and gentamicin (Gm), and to fusidic acid. Related to Figure 4.

	**MIC (μg/ml)**		
	**Km**	**Gm**	**Fusidic acid**
PcI	4.5	2	3000
fusA^{T680D}	9	20	12000
fusA^{K366N}	9	20	3000

Table S6. Strains used in this study. Related to STAR Methods.

Strain	***Genotype***	***Reference***
B. amyloliquefaciens FZB42	Wild type	*Bacillus* Genetic Stock Center (BGSC)
B. amyloliquefaciens FZB42	glpK::km	This study
B. amyloliquefaciens FZB42	Spontaneous mutant. $glpK^{F38S,\ W52stop,\ G254A}$	This study
B. amyloliquefaciens FZB42	*Spontaneous mutant.* $glpK^{G147R,\ Q421R}$	This study
B. amyloliquefaciens FZB42	*Spontaneous mutant.* $glpK^{W355R}$	This study
B. amyloliquefaciens FZB42	*Spontaneous mutant.* $glpK^{S166L}$	This study
B. amyloliquefaciens FZB42	*Spontaneous mutant.* $glpK^{+}$	This study
B. amyloliquefaciens FZB42	*Spontaneous mutant.* $glpK^{G162R,\ W485stop}$	This study
B. amyloliquefaciens FZB42	*Spontaneous mutant.* $glpK^{Q246-,\ G265S}$	This study
B. amyloliquefaciens FZB42	Wild type, amyE:: Pveg-yfp	This study
B. amyloliquefaciens FZB42	ΔfenA	*Bacillus* Genetic Stock Center (BGSC)
B. amyloliquefaciens FZB42	ΔbaeJ	BGSC
B. amyloliquefaciens FZB42	ΔbmyA	BGSC
B. amyloliquefaciens FZB42	Δdfn	BGSC

B. amyloliquefaciens FZB42	ΔsrfA	BGSC
P. chlororaphis PCL1606	Wild type	(Cazorla et al., 2006)
P. chlororaphis PCL1606	ΔPCL1606_RS10280 (PvdO)	This study
P. chlororaphis PCL1606	ΔPCL1606_RS14085 (AcsD)	This study
P. chlororaphis PCL1606	ΔPCL1606_RS13600 (PchC)	This study
P. chlororaphis PCL1606	ΔPCL1606_RS05710	This study
P. chlororaphis PCL1606	ΔPCL1606_RS13355	This study
P. chlororaphis PCL1606	ΔPCL1606_RS16180	This study
P. chlororaphis PCL1606	ΔPCL1606_RS15365	This study
P. chlororaphis PCL1606	ΔPCL1606_RS16050	This study
P. chlororaphis PCL1606	ΔPCL1606_RS23355	This study
P. chlororaphis PCL1606	ΔPCL1606_RS08425	This study
P. chlororaphis PCL1606	ΔPCL1606_RS13600 (PchC), ΔPCL1606_RS14085 (AcsD), ΔPCL1606_RS10280 (PvdO)	This study
P. chlororaphis PCL1606	ΔPCL1606_RS13600 (PchC), ΔPCL1606_RS10280 (PvdO)	This study
P. chlororaphis PCL1606	ΔPCL1606_RS10170-RS10180 (DarA-DarB-DarC)	This study
P. chlororaphis PCL1606	ΔPCL1606_RS10170-RS10180 (DarA-DarB-DarC), ΔPCL1606_RS10280 (PvdO)	This study
P. chlororaphis PCL1606	Spontaneous mutant. *fusA*^{T680D}	This study
P. chlororaphis PCL1606	Spontaneous mutant. *fusA*^{T680D}	This study
P. chlororaphis PCL1606	Spontaneous mutant. *fusA*^{K366N}	This study

Table S7. Oligonucleotides used in this study. Related to STAR Methods.

Name	Sequence
T2SS_up_Fwd	agtatagggataacagggtaatctgcaggacccggacatcatcatgatcggc
T2SS_up_Rev	cgataacggttcacaggcgtcccgggtcacacggagga
T2SS_down_Fwd	cccgggacgcctgtgaaccgttatcgctacgaagccgc
T2SS_down_Rev	agaggatccccgggtaccgagctcgcgtgaggatcttgccacgcagggtg
RS05710_up_Fwd	tctgaattcgagctcggtacccgggaccaccggcggcataagttgtcgtaatt
RS05710_up_Rev	tgcagctgcatgagtcccggaaggggccttcgaccata
RS05710_down_Fwd	ccccttccgggactcatgcagctgcaattcggagccga
RS05710_down_Rev	gcatgcctgcaggtcgactctagagcagctgggtgcggatgaagtgggaaaag
UP_RS14085_fwd	agtatagggataacagggtaatctgcgcgggctgggcctgatgctgggca
UP_RS14085_rev	ggtcgccgcgccaaaaggctccacagtagccatgcgatactggccacg
Down_RS14085_fwd	tgtggagccttttggcgcggcgacctgccgctgctcga
Down_RS14085_rev	agaggatccccgggtaccgagctcgcaactgagtgcgcagacgcaccacccgc
RS13600-up_fwd	agtatagggataacagggtaatctgcgatgaggtgaactcgccgtcattg
RS13600-up_rev	aagggtttttccgggatctcctatttgtagaaataattggctacaaatg
RS13600-DOWN_fwd	aataggagatcccggaaaaacccttccatgaaaacccctgac
RS13600-DOWN_rev	agaggatccccgggtaccgagctcgagaacaggctttcggcgtggggctg
UP_RS10280_Fwd	agtatagggataacagggtaatctgtctgcgcccgcagcacggagctcaa
UP_RS10280_Rev	ggcggtgcgggggggtcgtctcgaaggttggaaaaggtggatcgc
DOWN_RS10280_Fwd	ttcgagacgacccccccgcaccgccccacgccccgga
DOWN_RS10280_Rev	Agaggatccccgggtaccgagctcgggcagcggctacagatgcgagg cgttaacacgacatctgtaggagc
UP_RS10170_Fwd	tctgaattcgagctcggtacccggggtcgaaagagcgggcgccacgatgc
UP_RS10170_Rev	acaggaccggcgaaatacggggaccttttgctttacaaccacaaag
DOWN_RS10170_Fwd	ggtccccgtatttcgccggtcctgtgaagatgcggccg
DOWN_RS10170_Rev	gcatgcctgcaggtcgactctagagctatgtcacctcggtgtgcaccggc
RS15365_UP_Fwd	tctgaattcgagctcggtacccggggagagcgcaatagagcagccaggac
RS15365_UP_Rev	tggcctcccttaatgacgctccataaattaatcctacagtgag
RS15365_Down_Fwd	tatggagcgtcattaagggaggccaggcagcggccgtc
RS15365_Down_Rev	gcatgcctgcaggtcgactctagagtcagcagcctatccatgggagtgaagaccag
bact_RS23355_UP_fwd	tctgaattcgagctcggtacccgggcaggccggtgtgcgtcgtgctgttg
bact_RS23355_UP_rev	cggcttgggtatcgacactcaggcggacggtggattac
bact_RS23355_DOWN_fwd	cgcctgagtgtcgatacccaagccgccttcgccagcgc
bact_RS23355_DOWN_rev	gcatgcctgcaggtcgactctagagggcccatagcgcgaccactggatgag
GlpK_up_Fwd	tctgcagacgcgtcgacgtcatatgtttagatatccacctcggtcaattaaaag
GlpK_up_Rev	aaatggttcgctggatgccgctctcctttttaaatatattc
Km_GlpK_Fwd	gagagcggcatccagcgaaccatttgaggtgataggtaag
Km_GlpK_Rev	cacatttttatccgatacaaattcctcgtaggcgctc
GlpK_down_Fwd	ggaatttgtatcggataaaaatgtggtatactgaaaacaagttaatag
GlpK_down_Rev	tccagcctcgcgtcgggcgatatcgtagagccgtatctgatggctaactg
RS16180_UP_fwd	attcgagctcggtacccgggaagtgccgaggccgaaggaaggaat
RS16180_UP_rev	cgacaacttctgaatcattccaagttctctagttgcgaaaaac
RS16180_DOWN_fwd	gaatgattcagaagttgtcgttggcgtgcagcgcc

RS16180_DOWN_rev	cctgcaggtcgactctagaggttgattccctcttataaccaccgttttctactgg
RS08425_UP_fwd	tctagagtcgacctgcaggcatgcactgatgcgatcggcctcggcccccca
RS08425_UP_rev	tgttgccctgaggcaactctcctgctaccacgctgttttgaatgaac
RS08425_DOWN_fwd	gcaggagagttgcctcagggcaacaccccggctgcccc
RS08425_DOWN_rev	aaaaaagaatatataaggcttttaattccccaccaggtcgaaaccggtcagg
